# Development of 5‘ LTR DNA methylation of latent HIV-1 provirus in cell line models and in long-term-infected individuals

**DOI:** 10.1186/s13148-016-0185-6

**Published:** 2016-02-19

**Authors:** Kateřina Trejbalová, Denisa Kovářová, Jana Blažková, Ladislav Machala, David Jilich, Jan Weber, Dana Kučerová, Ondřej Vencálek, Ivan Hirsch, Jiří Hejnar

**Affiliations:** Institute of Molecular Genetics, Academy of Sciences of the Czech Republic, Vídeňská 1083, CZ-14220 Prague 4, Czech Republic; Department of Infectious Diseases, Third Faculty of Medicine, Charles University and Hospital Na Bulovce in Prague, Budínova 67/2, CZ-18081 Prague 8, Czech Republic; Department of Infectious, Tropical and Parasitic Diseases, First Faculty of Medicine, Charles University in Prague and Hospital Na Bulovce, Budínova 67/ 2, CZ-18081 Prague 8, Czech Republic; Institute of Organic Chemistry and Biochemistry, Academy of Sciences of the Czech Republic, Flemingovo nám. 2, CZ-16610 Prague 6, Czech Republic; Department of Mathematical Analysis and Applications of Mathematics, Faculty of Science of the Palacky University in Olomouc, Olomouc, CZ-77146 Czech Republic; Faculty of Science, Department of Genetics and Microbiology, Charles University in Prague, Viničná 5, CZ-12844 Prague 2, Czech Republic; Inserm, Centre de Recherche en Cancérologie de Marseille (CRCM), F-13273 Marseille, France; Institut Paoli-Calmettes, F-13009 Marseille, France; Aix-Marseille Univ., F-13284 Marseille, France; CNRS, UMR7258, CRCM, F-13009 Marseille, France

**Keywords:** HIV-1, Latent reservoir, DNA methylation, Chromatin conformation, Latent HIV-1 provirus reactivation, HIV-1-infected individuals

## Abstract

**Background:**

Human immunodeficiency virus type 1 (HIV-1) latency represents the major barrier to virus eradication in infected individuals because cells harboring latent HIV-1 provirus are not affected by current antiretroviral therapy (ART). We previously demonstrated that DNA methylation of HIV-1 long terminal repeat (5’ LTR) restricts HIV-1 reactivation and, together with chromatin conformation, represents an important mechanism of HIV-1 latency maintenance. Here, we explored the new issue of temporal development of DNA methylation in latent HIV-1 5’ LTR.

**Results:**

In the Jurkat CD4^+^ T cell model of latency, we showed that the stimulation of host cells contributed to de novo DNA methylation of the latent HIV-1 5’ LTR sequences. Consecutive stimulations of model CD4^+^ T cell line with TNF-α and PMA or with SAHA contributed to the progressive accumulation of 5’ LTR DNA methylation. Further, we showed that once established, the high DNA methylation level of the latent 5’ LTR in the cell line model was a stable epigenetic mark. Finally, we explored the development of 5’ LTR DNA methylation in the latent reservoir of HIV-1-infected individuals who were treated with ART. We detected low levels of 5’ LTR DNA methylation in the resting CD4^+^ T cells of the group of patients who were treated for up to 3 years. However, after long-term ART, we observed an accumulation of 5’ LTR DNA methylation in the latent reservoir. Importantly, within the latent reservoir of some long-term-treated individuals, we uncovered populations of proviral molecules with a high density of 5’ LTR CpG methylation.

**Conclusions:**

Our data showed the presence of 5’ LTR DNA methylation in the long-term reservoir of HIV-1-infected individuals and implied that the transient stimulation of cells harboring latent proviruses may contribute, at least in part, to the methylation of the HIV-1 promoter.

**Electronic supplementary material:**

The online version of this article (doi:10.1186/s13148-016-0185-6) contains supplementary material, which is available to authorized users.

## Background

A combination of antiretroviral drugs is the current treatment used to suppress human immunodeficiency virus type 1 (HIV-1) replication in infected patients. Nevertheless, the virus persists in the patient’s cellular reservoirs, where it is able to establish latent infection. The major HIV-1 reservoir is a small pool of latently infected resting memory CD4^+^ T cells carrying an integrated form of the viral genome [1–4]. The latent reservoir impedes HIV-1 eradication from the organism because under the non-productive state of infection, HIV-1 is refractory to immune surveillance and eradication by antiretroviral therapy (ART). Although the process of latency establishment is not yet clear, the maintenance of the latent reservoir determines the outcome of the therapy. Mutually cooperating mechanisms in HIV-1 transcriptional latency include transcriptional interference, insufficient levels of cellular transcriptional activators, chromatin conformation, DNA methylation, influence of the integration site, and fluctuations in Tat protein expression.

Once latency is established, repressive chromatin around the HIV-1 5’ LTR is critical for the maintenance of latency [5–13]. Considerable efforts are underway to employ agents that induce chromatin de-repression or activation of transcription for HIV-1 reactivation in vivo in cells of the latent reservoir. It is believed that infected cells in which HIV-1 latency is reversed will be killed by the immune response or viral cytopathic effects whereas new rounds of infection will be prevented by ART [14–16]. Histone deacetylase (HDAC) inhibitors, including valproic acid (VPA) and suberoylanilide hydroxamic acid (SAHA, Vorinostat), have been used in attempts to purge the latent reservoir, but without ultimate success [17–23]. One clinical study reported reduction of the latent viral reservoir size in infected individuals receiving ART and VPA treatment [24]. However, following clinical trials using VPA or SAHA failed to progressively reduce the frequency of infected resting CD4^+^ T cells [17, 18, 20–22, 25–31]. In agreement, it was demonstrated that HDAC inhibitors, including VPA and SAHA, do not induce HIV production in the latent viral reservoir of aviremic individuals in ex vivo system [32]. In addition, other HDAC inhibitors, panobinostat and romidepsin, stimulated HIV-1 expression in latently infected primary cells [33], in CD4^+^ T cells ex vivo and also in HIV-1-infected individuals in vivo [34, 35]. In summary, the results of clinical trials have demonstrated that the latent reservoir in vivo is reactivated only partially, and the latency maintenance mechanisms are therefore crucial targets for HIV-1-therapeutic strategies (reviewed in [36]).

Another factor that cooperates with chromatin structure in HIV-1 latency maintenance is DNA methylation. A study by Kauder et al. identified two CpG islands flanking the HIV-1 transcription start site, the first CpG island located in the U3 region, the second CpG island overlapping the last nucleotide of the U5 region and 189 nucleotides downstream of the HIV-1 5’ LTR [37]. Importantly, we and others have suggested that the DNA methylation of HIV-1 promoter/enhancer sequences is not involved in the establishment of latency but rather plays an important role in its maintenance [29, 37, 38]. We have shown previously in an in vitro model of HIV-1 latency and in a latent reservoir of HIV-1-infected patients that CpG methylation of the HIV-1 5’ LTR is an additional epigenetic restriction mechanism that controls the resistance of latent HIV-1 to reactivation signals and thus determines the stability of HIV-1 latency. However, we have shown that methylated proviruses can be reactivated to some extent using inhibitors of histone deacetylases and inhibitors of DNA methylation [29]. In this context, the DNA methylation levels of HIV-1 5’ LTR in the latent reservoir in vivo are highly important.

The methylation status of the 5’ LTR in patients was addressed in several studies with different outcomes. In our previous study, we analyzed the memory CD4^+^ T cells of six aviremic HIV-1-infected individuals who were treated for 11.5 years (median), and we detected 19 to 100 % CpG methylation in the 5’ LTR [29]. In the following studies, only 1 and 2 % of methylated CpGs in HIV-1 5’ LTR were detected in the resting CD4^+^ T cells of patients who were treated for 2.9 and 5 years, respectively [39, 40]. Moreover, the peripheral blood mononuclear cells (PBMCs) of long-term non-progressors displayed 0 and 5 % methylated CpGs in the HIV-1 5’ LTR [41, 42]. Overall, the assessments of the HIV-1 5’ LTR methylation levels in the latent reservoir of ART-treated HIV-1-infected individuals remain controversial and require further exploration.

In this study, we focused on the development of 5’ LTR DNA methylation in latent HIV-1. To explore this phenomenon, we took advantage of the latency cell line model that was validated in our previous study. We employed two Jurkat-derived cellular clones, H12 and 2D12, which harbor a latent HIV-1 “mini-provirus” (LTR-Tat-IRES-EGFP-LTR) whose reactivation can be monitored according to the percentage of EGFP-positive cells [29]. Both clones used in the previous and present study contained an identical and genetically unaltered single proviral copy that was integrated at the same site of the human genome within the intron of *ubiquilin* 1 gene. As we had shown previously, clone H12 displayed a low level of HIV-1 5’ LTR DNA methylation of the first CpG island (7 %), and the latent provirus was easily reactivated by various latency-reversing agents [29]. In contrast, clone 2D12 displayed a high level of 5’ LTR DNA methylation of the first CpG island (95 %), and the latent provirus was resistant to reactivation [29]. Importantly, the 2D12 clone was derived from H12 cells by mitogenic phorbol-12-myristate13-acetate (PMA) and tumor necrosis factor-α (TNF-α) stimulation and the subsequent selection of EGFP-negative subclones [29]. We showed that DNA methylation in the HIV-1 5’ LTR accumulated in the course of cell line stimulation by NF-κB inducers and selection of EGFP-negative cells.

To study the temporal development of DNA methylation of HIV-1 promoter we investigated whether the stimulation of Jurkat-derived latency model cell line harboring the HIV-1 provirus can induce DNA methylation of the 5’ LTR. We showed in this model that repeated transient stimulations of cells assisted de novo 5’ LTR DNA methylation of the latent HIV-1 provirus. However, the high DNA methylation level of the latent 5’ LTR was a stable epigenetic mark. Finally, we measured 5’ LTR DNA methylation in the latent reservoir of HIV-1-infected individuals who were treated for various periods of time. We demonstrated accumulation of DNA methylation in HIV-1 5’ LTR in the latent reservoir of HIV-1-infected individuals with a long history of ART. Our data showed that although HIV-1 5’ LTR methylation in the resting CD4^+^ T cells of HIV-1-infected individuals was a rare event, it increased with the time of reservoir persistence. Our results suggest that transient cellular stimulations may contribute, at least partially, to increase of 5’ LTR DNA methylation in the HIV-1 latent reservoir and, therefore, may contribute to the reservoir stability.

## Results

### Cellular stimulation contributed to de novo DNA methylation of the proviral 5’ LTR in the cell line model

**Fig. 1:**
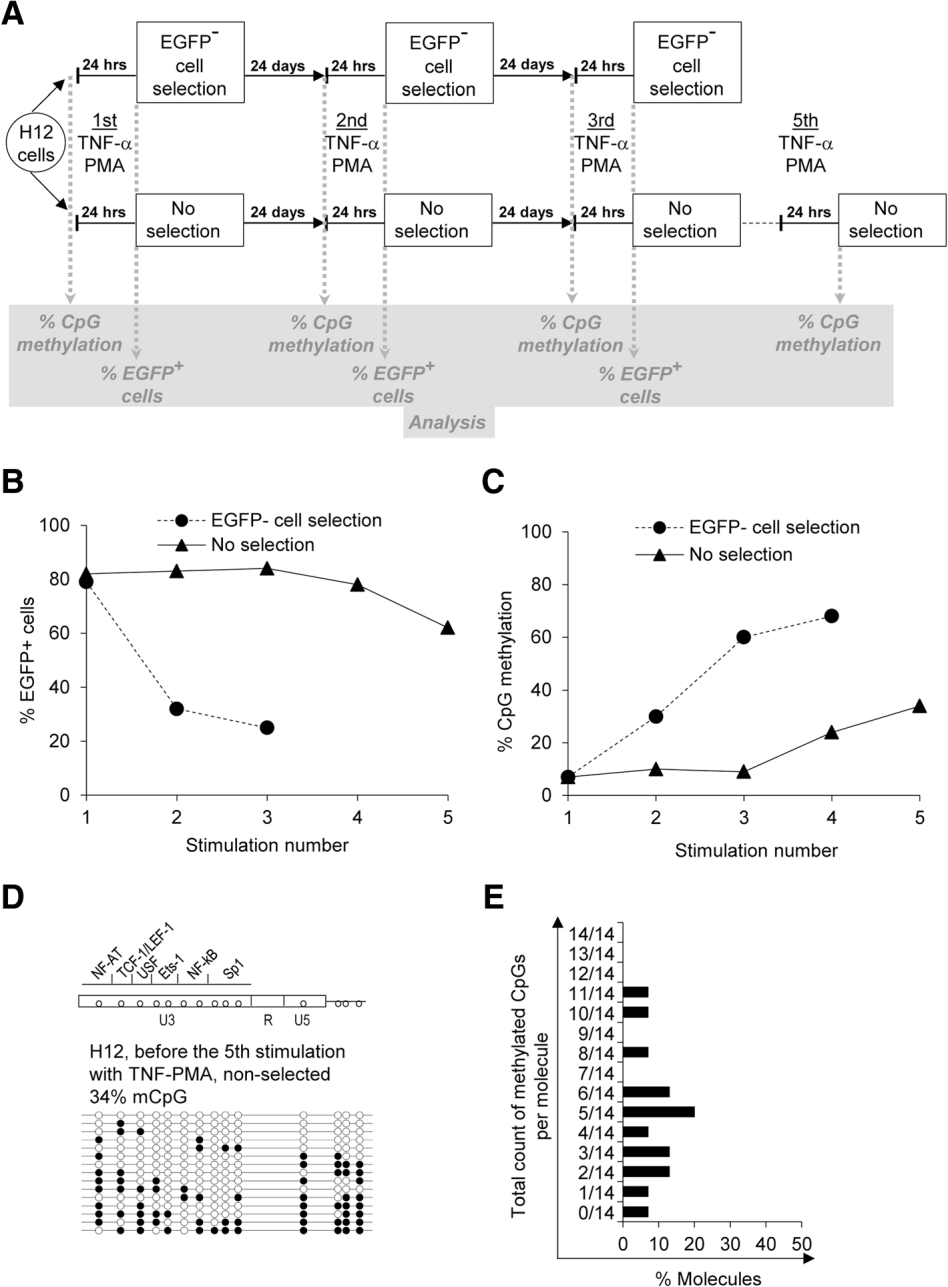
Cellular stimulation contributes to de novo DNA methylation of proviral 5’ LTR in the H12 cell line. **a** Flowchart of repeated stimulations of the H12 cell line with TNF-α and PMA performed with or without the selection of EGFP-negative cells by FACS-sorting. At the time of each stimulation, HIV-1 provirus reactivation after 24-h of TNF-α and PMA treatment was assessed according to the percentage of EGFP-positive cells. Bisulfite sequence determined the level of 5’ LTR CpG methylation. Bisulfite sequencing of the 5’ LTR was performed at 24 days after each stimulation, when the cells were restored to the non-stimulated, steady state. We assume the level of 5’ LTR DNA methylation controls the reactivation efficiency, however, the changes in 5’ LTR DNA methylation levels are not instant. Therefore, the levels of 5’ LTR methylation are determined immediately before the next stimulation cycle, e.g., the % of the 5’ LTR methylation depicted at the stimulation number 2 (Fig. 1c) is determined immediately before the 2^nd^ stimulation cycle, when the cells after the first stimulation re-established the non-stimulated, basal phenotype and displayed only basal levels of EGFP and CD69. Three cycles of cellular stimulations followed by the selection of EGFP-negative cells by FACS-sorting were performed, including three measurements of % EGFP-positive cells and four determinations of % CpG methylation. The last % CpG methylation was measured after the third stimulation, when the cells restored the non-stimulated steady state. Five cycles of cellular stimulations without selection of EGFP-negative cells were performed, including five measurements of % EGFP-positive cells and five determinations of % CpG methylation. The last % CpG methylation was measured 24 days after the fourth stimulation when the cells restored non-stimulated steady state. (The % CpG methylation 24 days after the fifth stimulation was not determined). **b** Latent HIV-1 provirus reactivation in the H12 cell line after repeated stimulations of cells. The H12 cell line was stimulated with TNF-α and PMA for 24 h, and the percentage of EGFP-positive cells was determined by FACS. The number of successive stimulations is depicted on the *x*-axis, and the percentage of EGFP-positive cells is depicted on the *y*-axis. The solid line shows activations without selection of EGFP-negative cells whereas the dashed line indicates activations followed by selection of EGFP-negative cells. **c** Latent HIV-1 provirus CpG methylation levels in the H12 5’ LTR sequences after repeated stimulations of cells. The percentage of methylated CpGs in the 5’ LTR was determined by bisulfite sequencing. The number of successive stimulations is depicted on the *x*-axis whereas the mean percentage of methylated CpGs is depicted on the *y*-axis. The solid line shows activations without selection of EGFP-negative cells, and the dashed line indicates activations followed by selection of EGFP-negative cells. The last point of the solid line applies to Fig. 1de. **d** The CpG methylation profile of the 5’ LTR in non-stimulated H12 cells before the fifth cycle of 24-h stimulation with TNF-α and PMA. The cycles of cellular stimulations were performed without selection of EGFP-negative cells. A schematic representation of the CpG dinucleotide distribution in 5’ LTR of the pEV731 vector together with the corresponding transcription factor binding sites is shown. Analysis of the promoter molecules is shown as a linear array of open circles representing non-methylated CpG residues and closed circles representing methylated CpG residues. Each line represents one sequenced molecule of the 5’ LTR. The methylation level is presented as a mean percentage of methylated CpGs (mCpGs) in HIV-1 promoters. **e** Distribution of the 5’ LTR molecules according to the proportion of methylated CpGs contained within. Non-stimulated H12 cells before the fifth cycle of 24-h stimulation with TNF-α and PMA are depicted. The cycles of cellular stimulations were performed without selection of EGFP-negative cells. The percentage of the respective molecules is shown on the *x*-axis. The total counts of methylated CpGs per molecule are shown on the *y*-axis

The accumulation of highly methylated latent proviral copies observed during consecutive cycles of provirus reactivation and negative selection could be explained either by the selection of preexisting non-reactivated methylated proviruses or by de novo proviral 5’ LTR DNA methylation induced in the process of TNF-α and PMA-mediated cell stimulations. To distinguish between these two mechanisms of provirus 5’ LTR methylation, we performed parallel repeated stimulations of the H12 cell line with or without the subsequent selection of EGFP-negative cells. At the time of each stimulation, we assessed HIV-1 provirus reactivation after TNF-α and PMA treatment according to the percentage of EGFP-positive cells. We also performed bisulfite sequencing of the 5’ LTR at 24 days after each stimulation, when the cells were restored to the non-stimulated, steady state. Our methylation analysis throughout the study concerned predominantly the first CpG island positioned upstream of the transcription start site [37]. A flowchart of the experiment is provided in Fig. 1a.

First, we reproduced the previously mentioned experiment using FACS-sorting of the EGFP-negative pool of H12 cells that contained non-activated latent provirus. After the first 24-h stimulation of the H12 cell line with TNF-α and PMA, the HIV-1 latent provirus was reactivated in 79 % of the cells. We sorted the remaining 21 % of EGFP-negative cells and let the cells restore to their non-stimulated steady state. We then repeated the stimulation with TNF-α and PMA and sorted the EGFP-negative cells (Fig. 1a). After the third cycle of stimulation, the latent provirus was reactivated in only 25 % of the cells (Fig. 1b). Simultaneously, the 5’ LTR methylation of the latent provirus as measured by bisulfite sequencing increased from 7 % of methylated CpGs before the first stimulation and sorting to 68 % after the third cycle of stimulation (Fig. 1c).

In parallel, we performed a series of consecutive stimulations of H12 cells without sorting the EGFP-negative cells. After the first 24-h stimulation of H12 cells with TNF-α and PMA, the HIV-1 latent provirus was reactivated in 82 % of the cells. We let the entire population of cells containing both reactivated and non-activated HIV-1 proviruses restore to the latent EGFP-negative steady state. We repeated this procedure five times (Fig. 1a). After the fifth cycle of stimulation, the latent provirus was reactivated only in 68 % of the cells (Fig. 1b). Simultaneously, the 5’ LTR methylation of the latent provirus increased from 7 % of methylated CpGs before the first stimulation to 34 % before the fifth stimulation (Fig. 1c). The results showed that accumulation of 5’ LTR DNA methylation did not require selection of EGFP-negative cells. To gain deeper insight into the mechanism of 5’ LTR DNA methylation accumulation we analyzed the individual 5’ LTR molecules within the methylation profile of the H12 cells before the fifth stimulation showing the average level of 34 % methylation (Fig. 1d). The majority of individual 5’ LTR molecules contained less than 50 % DNA methylation, arguing against the selection of preexisting non-reactivated methylated molecules (Fig. 1e). In a parallel culture of non-stimulated H12 cells, the hypomethylated status of the HIV-1 promoter remained stable (7 %). Our results noted that cellular stimulation with TNF-α and PMA supported de novo CpG methylation in the 5’ LTR of both reactivated and non-activated proviruses. Repeated cellular stimulations contributed, at least in part, to the progressive accumulation of DNA methylation in the latent HIV-1 promoter.

In contrast to progressive methylation of the initially hypomethylated latent promoter, the DNA methylation of highly methylated 2D12 5’ LTR remained stable after consecutive activations (Additional file 1: Figure S1A). We used the same repeated cycles of the 2D12 cells stimulation with TNFα and PMA. Surprisingly, we did not observe any continuous increase in the percentage of EGFP-positive 2D12 cells during this experiment, even after the sorting of EGFP-positive cells (Additional file 1: Figure S1B). In accordance, we did not detect decrease of 5’ LTR DNA methylation levels. Repeated cycles of cellular stimulation by NF-κB inducers did not alter the hypermethylated status of HIV-1 5’ LTR in the 2D12 cells.

Thus, we demonstrated that repeated stimulation cycles of Jurkat-derived CD4^+^ T cells harboring the latent HIV-1 provirus contributed to de novo CpG methylation within the 5’ LTR. The density of CpG methylation in the latent HIV-1 promoter increased gradually with the number of cell stimulation cycles even without the selection of EGFP-negative cells, although at a lower rate than in the selected population. The increase in the 5’ LTR DNA methylation level correlated with a decreased reactivation capacity of the latent provirus by NF-κB inducers. Furthermore, once established, the high DNA methylation level of the HIV-1 5’ LTR remained stable and resistant to demethylation.

### Both TNF-α-PMA and SAHA stimulation increased DNA methylation of the latent HIV-1 5’ LTR in the model cell line

**Fig. 2:**
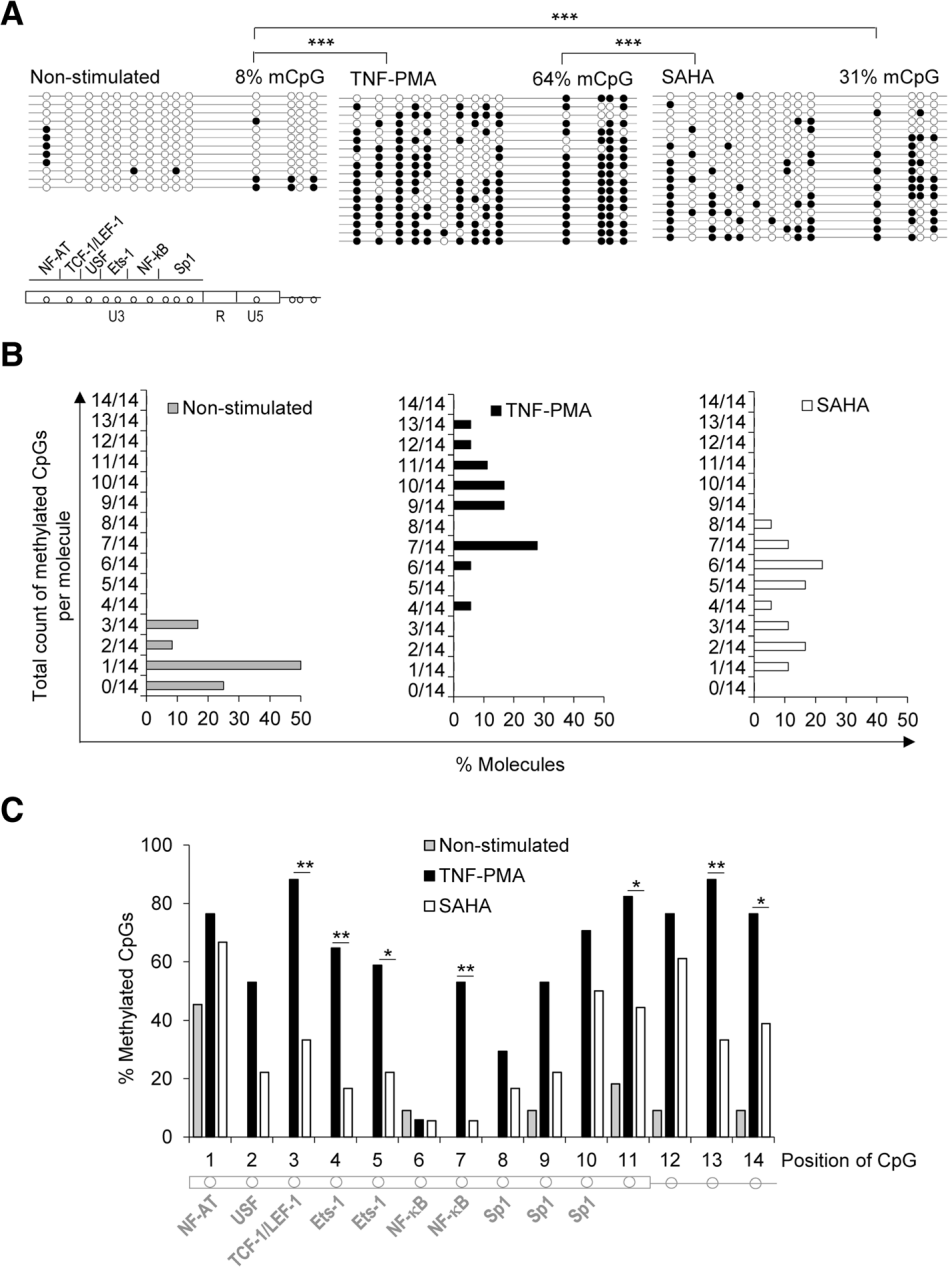
Both TNF-α-PMA and SAHA stimulation increase DNA methylation of the latent HIV-1 5’ LTR in the H12 cell line. **a** The CpG methylation profiles of the 5’ LTR in non-stimulated H12 cells and EGFP-negative H12 cells selected after a 24-h stimulation with TNF-α and PMA or with SAHA. A schematic representation of CpG dinucleotide distribution in the 5’ LTR of the pEV731 vector together with the corresponding transcription factor binding sites is shown. An analysis of the promoter molecules is shown as a linear array of open circles representing non-methylated CpG residues and closed circles representing methylated CpG residues. Each line represents one sequenced molecule of the 5’ LTR. The methylation levels are presented as a mean percentage of methylated CpGs (mCpGs) in HIV-1 promoters. The *p* values were calculated by the non-parametric Kruskal-Wallis test. Significance *** was assigned for *p* values <0.001. **b** Distribution of the 5’ LTR molecules according to the proportion of methylated CpGs contained within. Non-stimulated H12 cells and EGFP-negative H12 cells selected after a 24-h stimulation with TNF-α and PMA or with SAHA are depicted. The percentage of respective molecules is shown on the *x*-axis. The total counts of methylated CpGs per molecule are shown on the *y*-axis. **c** Percentage of methylated CpGs within individual positions of CpGs. Non-stimulated H12 cells and EGFP-negative H12 cells selected after a 24-h stimulation with TNF-α and PMA or with SAHA are depicted. Individual CpG positions are shown on the *x*-axis; a schematic representation of CpG dinucleotides in the 5’ LTR of the pEV731 vector together with the corresponding transcription factor binding sites is shown below the *x*-axis. The percentage of methylated CpGs is shown on the *y*-axis. The *p* values were calculated by Fisher’s exact test. Significance was assigned as follows: * for *p* values <0.05, ** for *p* values <0.01

Furthermore, we were interested in whether the stimulation of CD4^+^ T cells with different agents used for the reactivation of HIV-1 from latency induced similar effects on the latent provirus 5’ LTR methylation development. We analyzed TNF-α and PMA stimulation versus stimulation by SAHA. We stimulated the H12 cell line with TNF-α and PMA or with SAHA for 24 h and sorted out the EGFP-negative cells containing non-activated latent HIV-1 provirus. T cell activation by SAHA, which was measured by the expression of the early activation marker CD69, was substantially less efficient (35 %) in comparison to TNF-α and PMA stimulation (100 %, Additional file 2: Figure S2). However, reactivation of the latent HIV-1 provirus was efficient in both inductions. Stimulation by TNF-α and PMA led to EGFP expression in 90 % of H12 cells, whereas SAHA induced EGFP in 80 % of H12 cells (Additional file 2: Figure S2). We repeated the stimulation with TNF-α and PMA or with SAHA followed by selection of EGFP-negative cells twice. After the second series of stimulations, the 5’ LTR DNA methylation levels were significantly increased after both treatments compared to the original level of 8 %. Further, TNF-α and PMA induced significantly higher increase of the 5’ LTR DNA methylation (64 %) compared to SAHA (31 %), (*p* = 0.001; Fig. 2a).

In addition to the average DNA methylation, the level of CpG methylation of individual HIV-1 promoters could serve as a hallmark of proviral activity. In Fig. 2b, we showed the distribution of 5’ LTR molecules with increasing counts of methylated CpG dinucleotides within each molecule. In the non-stimulated culture of H12 cells, the HIV-1 promoter sequences with one methylated CpG were the most prevalent whereas 5’ LTR sequences with two or three methylated CpGs were rare (Fig. 2b). In contrast, in H12 cells stimulated with TNF-α and PMA, HIV-1 promoters with seven methylated CpGs prevailed, and many 5’ LTR molecules contained at least nine methylated CpGs out of 14 CpG dinucleotides (Fig. 2b). In H12 cells that were stimulated with SAHA, the distribution of methylated CpG was bimodal; this group of promoters showed a hypomethylated peak with one, two, or three methylated CpGs and another peak with five or six methylated CpGs, the maximal level being eight methylated CpGs in one molecule (Fig. 2b). In conclusion, a new category of 5’ LTR molecules with densely methylated CpGs were present in the TNF-α and PMA-stimulated EGFP-negative cell population in comparison with the non-stimulated cells.

Finally, we analyzed the patterns of 5’ LTR DNA methylation. The DNA methylation levels at the individual CpG dinucleotides (Fig. 2c) were significantly different at seven CpGs in TNF-α and PMA-stimulated H12 cells compared to SAHA-stimulated H12 cells. We observed an intriguing situation at the NF-κB binding site concerning CpG nos. 6 and 7. CpG no. 6 was the only one of 14 CpGs that did not display increased DNA methylation after any type of cell stimulation. DNA methylation at CpG no. 7 was significantly increased in latent proviruses after stimulation with TNF-α and PMA but not after stimulation with SAHA (*p* < 0.01).

In summary, we have shown that stimulation of CD4^+^ T cells with both TNF-α and PMA or SAHA followed by EGFP-negative cell selection induced a significant accumulation of DNA methylation at the 5’ LTR of the latent HIV-1 provirus. In accordance with more efficient cellular stimulation, TNF-α and PMA contributed to significantly higher DNA methylation of the 5’ LTR than the SAHA treatment.

### Hypermethylation of 5’ LTR DNA in the HIV-1 latency model cell line is maintained by DNMT1

Assuming the role of provirus 5’ LTR DNA methylation in the maintenance of HIV-1 latency, we explored the stability of HIV-1 5’ LTR hypermethylation in the 2D12 cell line. We knocked down the expression of DNA methyltransferase 1 (DNMT1) by siRNA (siDNMT1) and analyzed the effect of DNMT1 knockdown on the HIV-1 provirus reactivation by NF-κB inducers and its 5’ LTR DNA methylation level.

**Fig. 3:**
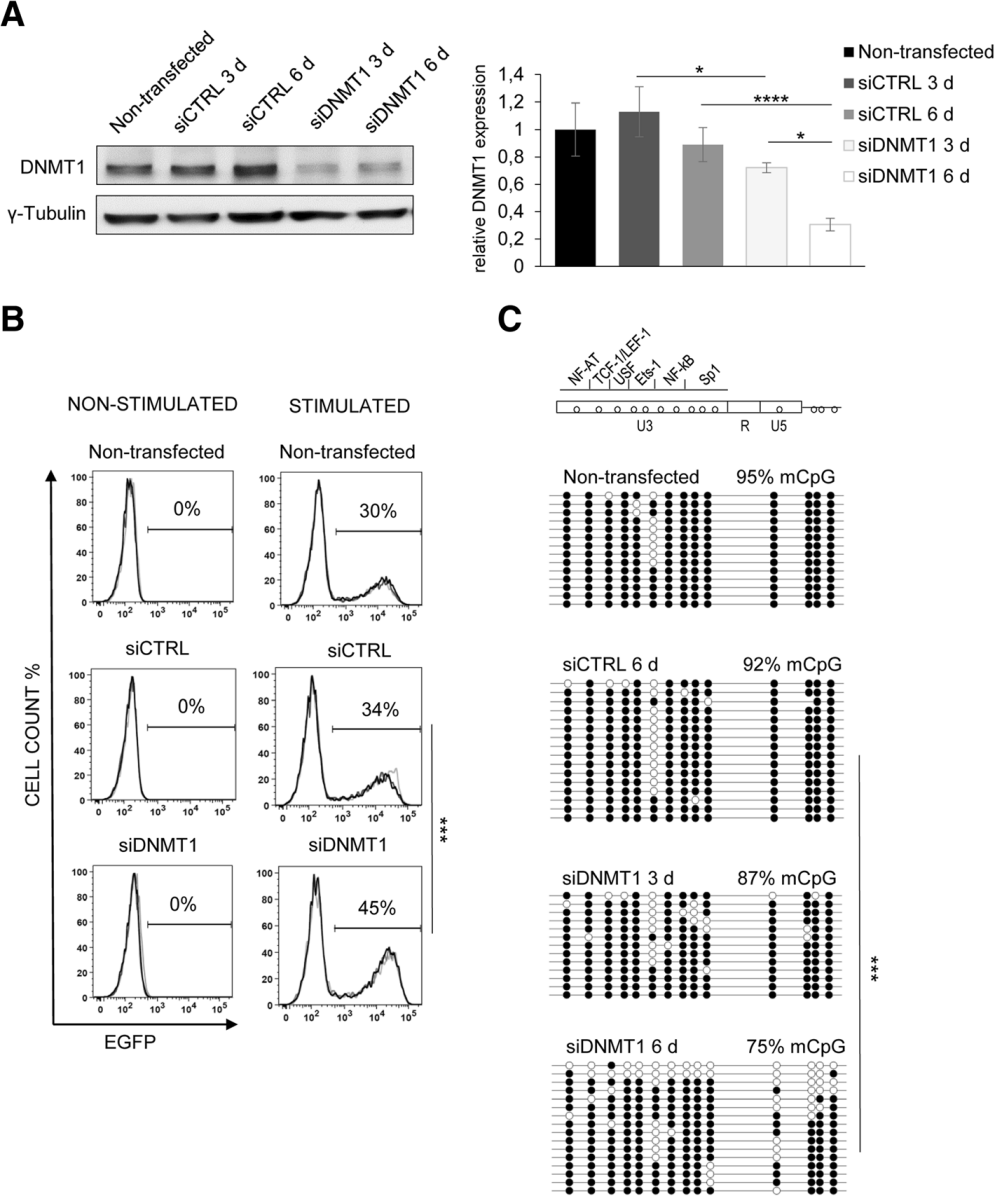
Hypermethylation of 5’ LTR DNA in the HIV-1 latency model cell line is maintained by DNMT1. **a** DNMT1 protein expression in 2D12 cell lines that were transfected with siCTRL and with siDNMT1 at days 3 and 6 after transfection as determined by the specific antibody is depicted on the left panel (*upper band*). Equal protein loading was verified by anti-γ-tubulin antibodies as depicted on the left panel (*lower band*). The DNMT1 relative mRNA levels at days 0 (control), 3, and 6 of siCTRL or siDNMT1 transfection in the 2D12 cell line as determined by qRT-PCR are shown in the right panel. mRNA expression was normalized to the expression of the RNA polymerase II, polypeptide A (*POLR2A*) housekeeping gene. The data are presented as the mean ± SD of technical triplicates. Three independent experiments were performed, representative example is shown. The *p* values were calculated by the non-paired Student’s *t* test. Significance * was assigned for *p* values <0.05. **b** Latent HIV-1 provirus reactivation levels after 6 days of DNMT1 knockdown. 2D12 cells were transfected with siCTRL or with siDNMT1. The left column of histograms represents analyses without cellular stimulation whereas the right column represents histograms after 24 h of TNF-α and PMA stimulation. Three FACS analyses of three independent experiments are overlaid; the mean percentage of EGFP-positive cells is shown above the bar. The *p* values were calculated by the non-paired Student’s *t* test. Significance *** was assigned for *p* values <0.001. **c** CpG methylation profile of 5’ LTR in non-stimulated 2D12 cells. The methylation profiles were obtained from cells transfected with siCTRL or those transfected for 3 and 6 days with siDNMT1. Methylation levels are presented as a mean percentage of methylated CpGs (mCpGs) in HIV-1 promoters. An analysis of promoter molecules is shown as a linear array of open circles representing non-methylated CpG residues and closed circles representing methylated CpG residues. Each line represents one sequenced molecule of the 5’ LTR. Mixture of genomic DNA isolated from three independent experiments was used for bisulfite analysis. The rectangle schematically represents the 5’ LTR regions U3, R, and U5 with individually analyzed CpG dinucleotides and transcription factor binding sites. The *p* values were calculated by the non-parametric Kruskal-Wallis test. Significance *** was assigned for *p* values <0.001

We observed the depletion of DNMT1 both at the protein and mRNA level 3 and 6 days after transfection (*p* < 0.05 for mRNA level; Fig. 3a). The DNMT1 knockdown did not activate latent HIV-1 provirus expression in the non-stimulated 2D12 cell line (0 % of EGFP-positive cells; Fig. 3b, left column); however, 6 days after the transfection, there was a more efficient provirus reactivation after stimulation by TNF-α and PMA in the DNMT1 knocked down cells compared to the 2D12 cells that were transfected with siCTRL (45 versus 34 %; *p* < 0.01; Fig. 3b, right column). Furthermore, the DNMT1 knockdown did not change the percentage of stimulated CD4^+^ T cells (97 % of CD69-positive cells), and the provirus was reactivated in CD69-positive cells (Additional file 3: Figure S3). The transfection of control siRNA (siCTRL) did not affect the provirus reactivation in either the non-stimulated cells or in the TNF-α- and PMA-stimulated 2D12 cells (Fig. 3b).

We estimated the changes in the 5’ LTR methylation level as a direct effect of the DNMT1 knockdown. Using bisulfite sequencing, no decrease in the 5’ LTR methylation was observed after siCTRL transfection. We detected a mild decrease of 5’ LTR CpG methylation in 2D12 cells after the DNMT1 knockdown. The 5’ LTR DNA methylation was reduced by 17 % 6 days after transfection of siDNMT1 (*p* < 0.001; Fig. 3c).

**Fig. 4:**
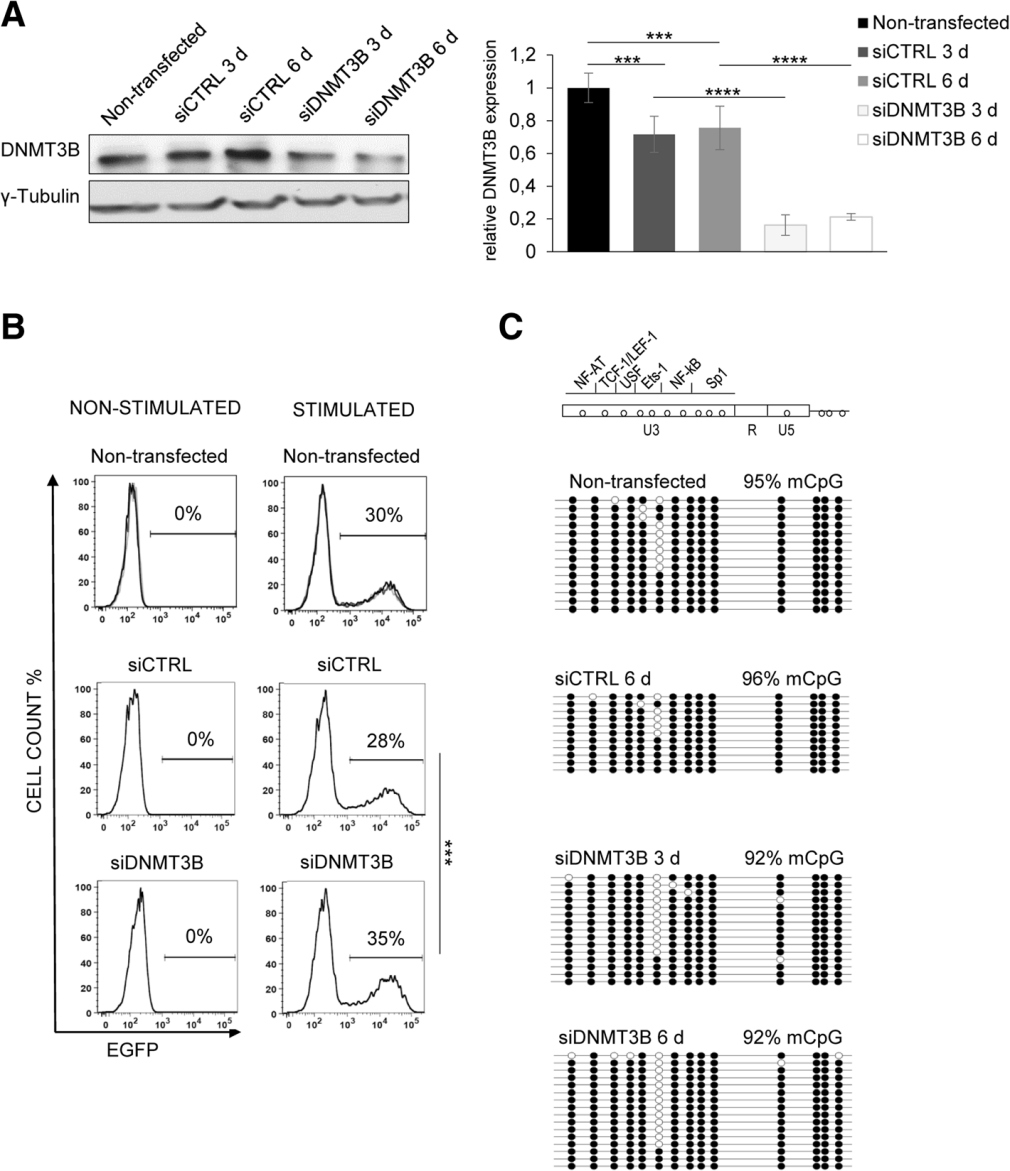
Hypermethylation of 5’ LTR DNA in the HIV-1 latency model cell line 2D12 is not maintainted by DNMT3B. **a** DNMT3B protein expression in 2D12 cell lines that were transfected with siCTRL and with siDNMT3B at days 3 and 6 after transfection as determined by the specific antibody is depicted on the left panel (*upper band*). Equal protein loading was verified by anti-γ-tubulin antibodies as depicted on the left panel (*lower band*). The DNMT3B relative mRNA levels at days 0 (control), 3, and 6 of siCTRL or siDNMT3B transfection in the 2D12 cell line as determined by qRT-PCR are shown in the right panel. mRNA expression was normalized to the expression of the RNA polymerase II, polypeptide A (*POLR2A*) housekeeping gene. The data are presented as the mean ± SD of technical triplicates. Three independent experiments were performed, representative example is shown. The *p* values were calculated by the non-paired Student’s *t* test. Significance *** was assigned for *p* values <0.001. **b** Latent HIV-1 provirus reactivation levels after 6 days of DNMT3B knockdown. 2D12 cells were transfected with siCTRL or with siDNMT3B. The left column of histograms represents analyses without cellular stimulation whereas the right column represents histograms after 24 h of TNF-α and PMA stimulation. Three FACS analyses of three independent experiments are overlaid; the mean percentage of EGFP-positive cells is shown above the bar. The *p* values were calculated by the non-paired Student’s *t* test. Significance *** was assigned for *p* values <0.001. **c** CpG methylation profile of 5’ LTR in non-stimulated 2D12 cells. The 5’ LTR methylation profiles were obtained from cells transfected with siCTRL or those transfected for 3 and 6 days with siDNMT3B. Methylation levels are presented as a mean percentage of methylated CpGs (mCpGs) in HIV-1 promoters. An analysis of promoter molecules is shown as a linear array of open circles representing non-methylated CpG residues and closed circles representing methylated CpG residues. Each line represents one sequenced molecule of the 5’ LTR. Mixture of genomic DNA isolated from three independent experiments was used for bisulfite analysis. The rectangle schematically represents the 5’ LTR regions U3, R, and U5 with individually analyzed CpG dinucleotides and transcription factor binding sites.

Next, we performed a knockdown of DNA methyltransferase 3B, DNMT3B, using siRNA (siDNMT3B). We verified the depletion of DNMT3B in the 2D12 cell line at both the mRNA and protein levels (Fig. 4a). The DNMT3B knockdown did not induce provirus reactivation. TNF-α and PMA stimulation slightly increased provirus reactivation in siDNMT3B-transfected 2D12 cells 6 days after transfection, by 7 % compared to siCTRL-transfected cells (Fig. 4b). We did not observe any decrease in 5’ LTR DNA methylation in the 2D12 cells with depleted DNMT3B 6 days after transfection (Fig. 4c).

These results show that the high methylation level of the latent 5’ LTR is partially reduced in cells with DNMT1 depletion. However, we observed no effect of DNMT3B depletion, suggesting a role for DNMT1 in the maintenance of 5’ LTR hypermethylation in the 2D12 model cell line. In accordance with our previous results, the reduction of the 5’ LTR methylation level of the provirus correlated with the increased reactivation capacity of the latent HIV-1 after activation with TNF-α and PMA.

### 5’ LTR DNA methylation is resistant to HDAC2 depletion in the HIV-1 latency model cell line

**Fig. 5:**
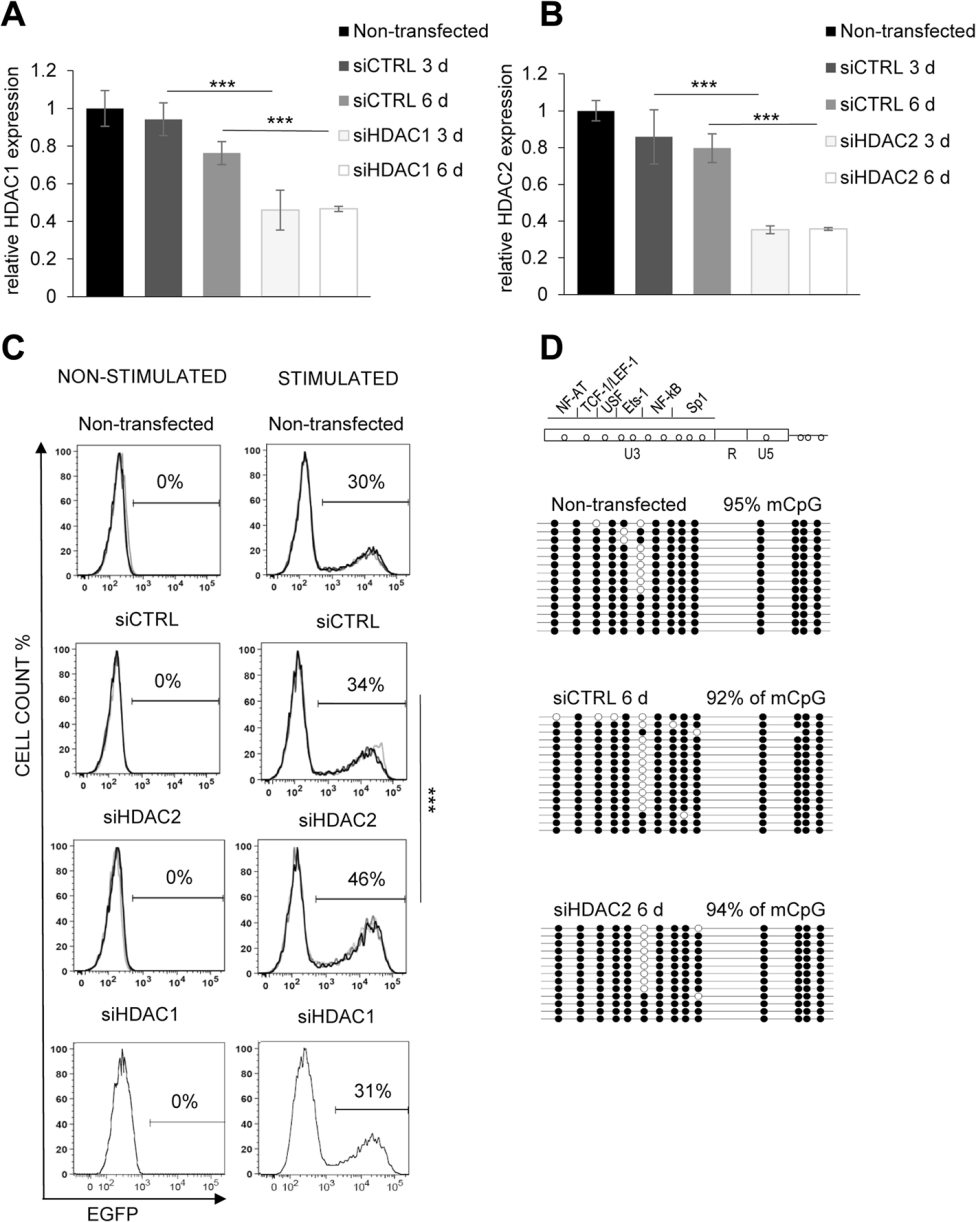
5’ LTR DNA methylation is resistant to HDAC depletion in the HIV-1 latency model cell line. **a** HDAC1 relative mRNA levels at days 0 (control), 3, and 6 after siCTRL or siHDAC1 transfection in the 2D12 cell line as determined by qRT-PCR. mRNA expression was normalized to the expression of the RNA polymerase II, polypeptide A (*POLR2A*) housekeeping gene. The data are presented as the mean ± SD of technical triplicates. Three independent experiments were performed, representative example is shown. The *p* values were calculated by the non-paired Student’s *t* test. Significance *** was assigned for *p* values <0.001. **b** HDAC2 relative mRNA levels at days 0 (control), 3, and 6 after siCTRL or siHDAC2 transfection in the 2D12 cell line determined by qRT-PCR. mRNA expression was normalized to the expression of the RNA polymerase II, polypeptide A (*POLR2A*) housekeeping gene. The data are presented as the mean ± SD of technical triplicates. Three independent experiments were performed, representative example is shown. The *p* values were calculated by the non-paired Student’s *t* test. Significance *** was assigned for *p* values <0.001. **c** Latent HIV-1 provirus reactivation levels after 6 days of HDAC2 and HDCA1 knockdown. 2D12 cells that were transfected with siCTRL or with siHDAC2 and siHDAC1 are shown, either without cellular stimulation (*left column*) or after 24 h of TNF-α and PMA stimulation (*right column*). Three FACS analyses of three biological replicates are overlaid; the mean percentage of EGFP-positive cells is shown on the *x*-axis, whereas the mean fluorescence intensity of EGFP is shown on the *y*-axis. The *p* values were calculated by the non-paired Student’s *t* test. Significance *** was assigned for *p* values <0.001. **d** CpG methylation profile of 5’ LTR in the non-stimulated 2D12 cell line. Latent HIV-1 provirus CpG methylation levels at the 5’ LTR sequences after 6 days of HDAC2 knockdown in the 2D12 cell line. Methylation levels are presented as a mean percentage of methylated CpGs (mCpGs) in HIV-1 promoters. An analysis of promoter molecules is shown as a linear array of open circles representing non-methylated CpG residues and closed circles representing methylated CpG residues. Each line represents one sequenced molecule of the 5’ LTR. Mixture of genomic DNA isolated from three independent experiments was used for bisulfite analysis. The rectangle schematically represents the 5’ LTR regions U3, R, and U5 with individually analyzed CpG dinucleotides and transcription factor binding sites

We described earlier that latency of the hypermethylated proviral promoter in 2D12 cells was associated with the repressive chromatin marks H3K9 me3 and H3K27 me3. In contrast, 2D12 provirus reactivation by NF-κB inducers was followed by an increase in H3 acetylation at the 5’ LTR [29]. We analyzed the reactivation capacity and 5’ LTR DNA methylation level of the 2D12 provirus after histone deacetylase (HDAC) knockdown by siRNAs. The expression of HDAC1 and HDAC2, which are prominent HDACs in the 2D12 cell line (Additional file 4: Figure S4), was targeted by siRNA. The expression of both HDAC1 (Fig. 5a) and HDAC2 (Fig. 5b) was significantly decreased at the mRNA level 3 and 6 days after siRNA transfection (*p* < 0.001).

The HDAC1 knockdown did not exert any effects on provirus reactivation in either the non-stimulated or in stimulated 2D12 cells (Fig. 5c). The HDAC2 knockdown did not induce latent provirus reactivation in non-stimulated 2D12 cells (Fig. 5c, left column). However, 6 days after transfection TNF-α and PMA stimulation reactivated the latent provirus in 46 % of 2D12 cells with knocked down HDAC2 compared to 34 % of siCTRL-transfected cells or 30 % of non-transfected 2D12 cells (*p* < 0.01; Fig. 5c, right column). We did not observe any significant decrease in the proviral 5’ LTR DNA methylation level after HDAC2 knockdown in the 2D12 cell line. The 2D12 cells that were transfected with siRNAs targeting HDAC2 displayed 94 % methylated CpGs in the 5’ LTR, which was comparable with the 92 % that was found in 2D12 cells transfected with control siRNA (Fig. 5d).

In accordance with our previous findings showing the importance of both DNA methylation and chromatin deacetylation for HIV-1 latency maintenance, the depletion of HDAC2 led to the partial reactivation of the HIV-1 provirus with a hypermethylated 5’ LTR after TNF-α and PMA stimulation. However, in our CD4^+^ T cell line model, we observed no effect of HDAC2 knockdown on the methylation level of the latent HIV-1 provirus 5’ LTR, which remained stable.

### DNA methylation of HIV-1 5’ LTR in the latent reservoir of ART-treated patients

**Fig. 6:**
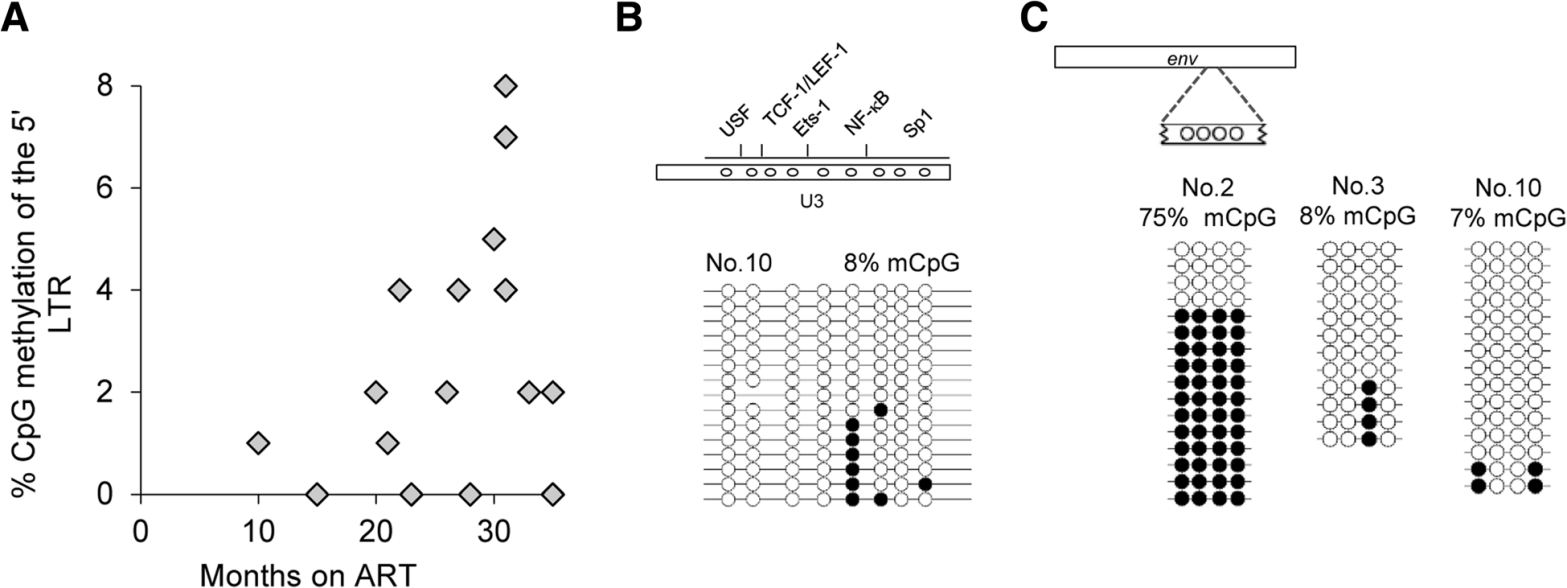
5’ LTR and *env* methylation levels of the HIV-1 provirus in the latent reservoir of infected patients who were treated for up to 3 years. **a** Levels of HIV-1 5’ LTR methylation in patients who were treated for up to 3 years. Each diamond represents one patient. The time (months) spent undergoing therapy is depicted on the *x*-axis whereas the percentage of methylated CpGs in the HIV-1 5’ LTR in the latent reservoir of infected patients is depicted on the *y*-axis. The methylation levels are presented as a mean percentage of methylated CpGs in HIV-1 promoters. Temporal increase in 5‘LTR DNA methylation was statistically significant (p < 0.05) as determined by standard significance test in loglinear model. **b** Illustrative example of the latent HIV-1 provirus CpG methylation profile at the 5’ LTR sequence of patient no. 10. The methylation level is presented as a mean percentage of methylated CpGs (mCpGs) in HIV-1 promoters. An analysis of promoter molecules is shown as a linear array of open circles representing non-methylated CpG residues and closed circles representing methylated CpG residues. Each line represents one sequenced molecule of the 5’ LTR. The rectangle schematically represents the 5’ LTR region U3 with individually analyzed CpG dinucleotides and transcription factor binding sites. **c** Levels of HIV-1 *env* methylation in selected patients who were treated for up to 3 years. The methylation levels are presented as a mean percentage of methylated CpGs (mCpGs) in HIV-1 env region. An analysis of *env* molecules is shown as a linear array of open circles representing non-methylated CpG residues and closed circles representing methylated CpG residues. Each line represents one sequenced *env* molecule. The rectangle schematically represents the analyzed *env* region

Next, we focused on HIV-1 5’ LTR DNA methylation development directly in HIV-1-infected individuals. To study the early development of HIV-1 5’ LTR methylation in the latent reservoir of resting CD4^+^ T cells, we enrolled a cohort of 15 HIV-1-infected individuals who were effectively treated by antiretroviral therapy for 9 to 35 months (median 27.5 months, Additional file 5: Table S1). We supposed that under effective ART, resting CD4^+^ T cells containing the HIV-1 provirus represented the major latent reservoir. We isolated the resting CD4^+^ T cells from patient PBMCs and analyzed the HIV-1 5’ LTR methylation levels by bisulfite sequencing (Additional file 6: Figure S5). We detected low levels of 5’ LTR methylation (median 2 % methylated CpGs), with a maximum of 8 % methylation of the 5’ LTR (Fig. 6a). Of note, we observed significant increase in 5’ LTR DNA methylation during prolonged therapy (*p* < 0.05; Fig. 6a; Additional file 5: Table S1).

The 5’ LTR methylation profile was exemplified by individual no. 10 with 8 % of 5’ LTR methylation (Fig. 6b). None of the analyzed patients contained more than two methylated CpGs in one 5’ LTR molecule. This indicated that highly methylated proviral molecules, which are expected to be resistant to reactivation, do not accumulate in the latent reservoir of patients who are treated for up to 3 years. The positions of the methylated CpGs within the 5’ LTR did not show any site-specific patterns. In some individuals, we observed polymorphisms of the 5’ LTR sequences including mutations of individual CpG dinucleotides. For example, all 5’ LTR sequences of patient no. 10 lacked the third CpG (Fig. 6b). Mutations in the 5’ LTR may generate defective proviruses. Such proviruses were shown to be preferentially methylated. However, we detected low methylation levels of proviruses with altered CpG dinucleotide patterns.

In addition, we determined CpG methylation within the envelope coding region comprising a CpG island that does not contribute to the regulation of viral transcription [39]. Three HIV-1-infected individuals displayed variable levels of DNA methylation ranging from 8 to 75 % CpG methylation in resting CD4^+^ T cells showing that analyzed HIV-1 DNA can be methylated (Fig. 6c). It also suggests that HIV-1 proviruses are subjected to the DNA methylation in infected individuals, but are protected within the region important for transcriptional regulation.

Taken together, and in agreement with previous reports, we found low methylation levels of HIV-1 5’ LTR in the latent reservoir of infected individuals who were treated for a maximal period of 35 months.

### DNA methylation of HIV-1 5’ LTR in the latent reservoir of patients with a long history of ART

**Fig. 7:**
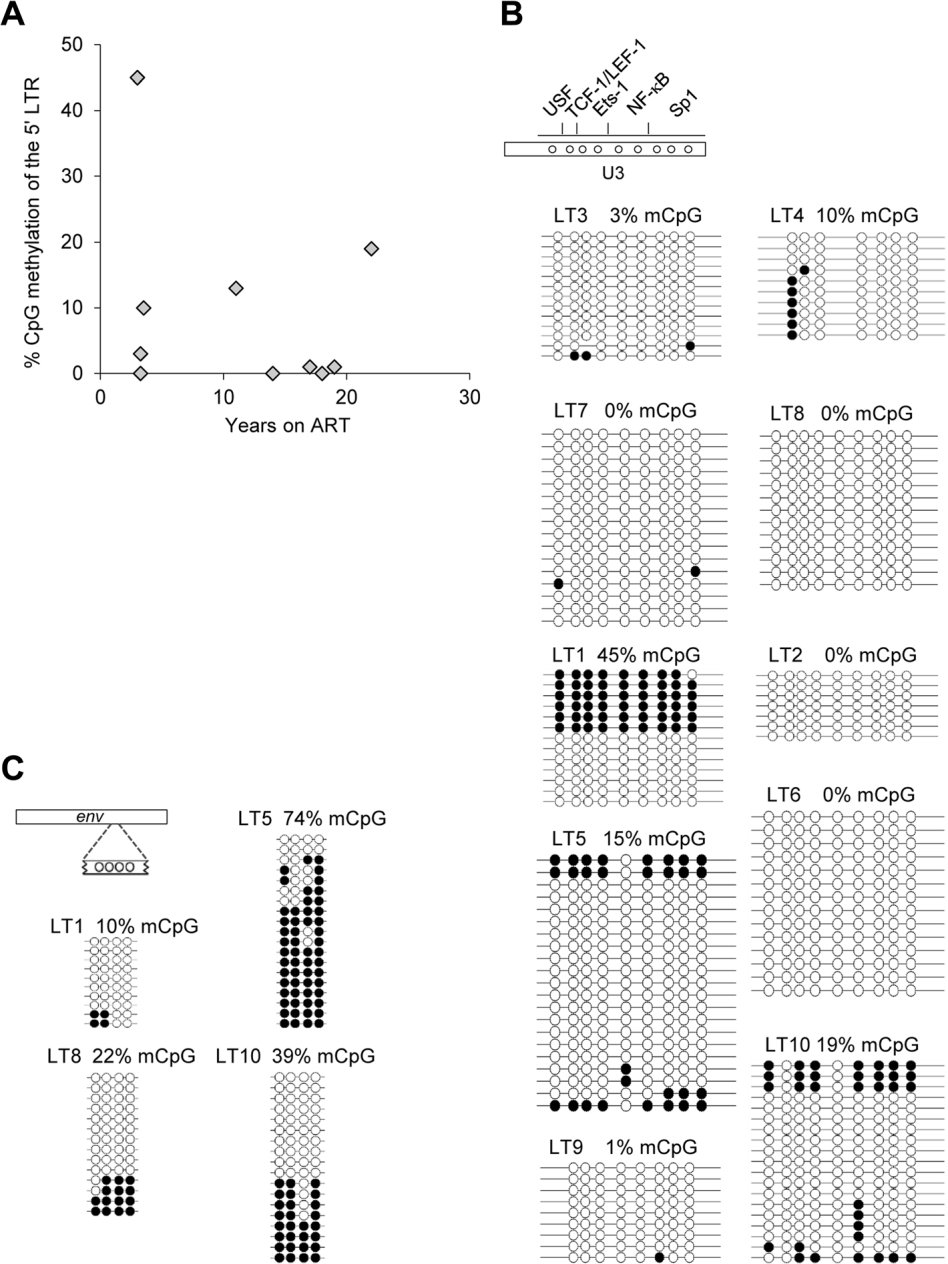
5’ LTR and *env* methylation levels of the HIV-1 provirus in the latent reservoir of long-term treated HIV-1-infected individuals. **a** The levels of HIV-1 5’ LTR methylation in long-term treated patients. Each diamond represents one patient. The time (years) spent undergoing therapy is depicted on the *x*-axis whereas the percentage of methylated CpGs in the 5’ LTR of HIV-1 in the latent reservoir of infected patients is depicted on the *y*-axis. The methylation levels are presented as a mean percentage of methylated CpGs in HIV-1 promoters. **b** Latent HIV-1 provirus CpG methylation profiles at the 5’ LTR sequences of long-term treated patients nos. LT1 to LT10. The methylation levels are presented as a mean percentage of methylated CpGs (mCpGs) in HIV-1 promoters. An analysis of promoter molecules is shown as a linear array of open circles representing non-methylated CpG residues and closed circles representing methylated CpG residues. Each line represents one sequenced molecule of the 5’ LTR. The rectangle schematically represents the 5’ LTR region U3 with a distribution of individually analyzed CpG dinucleotides and transcription factor binding sites. **c** Levels of HIV-1 *env* methylation in selected long-term-treated individuals. The methylation levels are presented as a mean percentage of methylated CpGs mCpGs) in HIV-1 *env* region. An analysis of *env* molecules is shown as a linear array of open circles representing non-methylated CpG residues and closed circles representing methylated CpG residues. Each line represents one sequenced *env* molecule. The rectangle schematically represents the analyzed *env* region.

To further explore the long-term (LT) development of 5’ LTR methylation in the latent reservoir of HIV-1-infected individuals, we enrolled another cohort of 10 patients who were treated for 3 to 22 years (median 12.5 years; Additional file 7: Table S2). The DNA methylation analysis of HIV-1 5’ LTR, which was performed in the same manner as in the first cohort, showed different levels of methylation ranging from 0 to 45 % methylated CpGs (Fig. 7a). The highest result, 45 % methylated CpGs, was found in a single patient (no. LT1); three other patients displayed 10, 15, and 19 % methylated CpGs, respectively, and six patients displayed between 0 and 3 % methylated CpGs in the 5’ LTR (Fig. 7b). In several individuals, we also analyzed the HIV-1 5’ LTR methylation levels in isolated memory CD4^+^ T cells. We obtained identical results for both memory and resting CD4^+^ T cell populations (data not shown). As in the group of patients treated for up to 3 years, the CpG methylation within the envelope coding region of four long-term-treated individuals displayed variable levels of CpG methylation ranging from 10 to 74 % (Fig. 7c). In the latent reservoir of three patients with high or moderate average 5’ LTR DNA methylation levels, we detected both heavily methylated and non-methylated 5’ LTR molecules within the same individual (patient nos. LT1, LT5, and LT10). Methylated 5’ LTR sequences of the long-term treated patients did not display large rearrangements or mutations of CpG dinucleotides (Fig. 7b). However, mutated 5’ LTR CpG dinucleotides were detected in 5’ LTR sequences with low methylation levels in patient nos. LT4 and LT9 (Fig. 7b). As for the previous cohort, our results do not correspond with the preferential methylation of defective 5’ LTRs of the HIV-1 provirus. Finally, in the highly methylated 5’ LTRs in patient nos. LT5 and LT10, we observed a lack of methylation at the CpG located within the NF-κB binding site which was identical with the hypomethylated CpG in the model cell lines. When we evaluated the 5’ LTR CpG methylation levels from all patients fused into one group, we found a statistically significant increase of the 5’ LTR DNA methylation levels (Additional file 8: Figure S6, *p* < 0,001). The percentage of methylated cytosines contained in the 5’ LTR arose by 7.5 % of its original value per year.

In summary, long-term ART contributed to the accumulation of CpG methylation in 5’ LTR in the HIV-1 latent reservoir. Furthermore, densely methylated 5’ LTR molecules could be found within the latent reservoirs of patients with a long history of ART. Our results in infected individuals are also supported by the progressive increase of 5’ LTR CpG methylation in the Jurkat cell line model.

## Discussion

Our results from the T cell model of HIV-1 latency showed that repeated transient stimulations of cells with TNF-α and PMA, which occurred via PKC and NF-κB and caused efficient T cell activation [43–45], contributed, at least partially, to de novo DNA methylation of the 5’ LTR. Furthermore, stimulation with SAHA, which did not cause global activation of resting T cells [32, 33], also supported accumulation of DNA methylation of the 5’ LTR, although at a lower rate than TNF-α and PMA stimulation. It should be noted, however, that in contrast to the reservoir of infected individuals, our Jurkat-derived model cell lines H12 and 2D12 harbored the HIV-1 mini-virus. Nevertheless, in our present work, we observed the long-term accumulation of 5’ LTR DNA methylation in the latent reservoir of HIV-1-infected individuals. In the group of 10 long-term-treated patients, we detected four individuals with 10 to 45 % methylated CpGs in the latent HIV-1 5’ LTR. We suggest that 5’ LTR DNA methylation may accumulate in rare proviral copies in the latent reservoir of HIV-1-infected individuals as a result of transient stimulations of reservoir cells.

Accumulation of 5’ LTR DNA methylation in vivo as a result of transient stimulations of reservoir cells could, paradoxically, be provoked by therapeutic reactivation approaches. However, exact mechanism of accumulation of DNA methylation in the latent reservoir of HIV-1-infected individuals is not clear. The high concentration of deamination-prone methylcytosines in the proviral 5’ LTR may lead to the higher mutation rates in the latent promoter. This mechanism may render the provirus defective for replication, or it may relieve transcriptional silencing [46].

Accumulation of the methylated 5’ LTR sequences in the stimulated H12 cells and in the long-term reservoir of HIV-1-infected individuals is compatible with de novo methylation of the HIV-1 promoter or with selective survival of cells bearing profoundly latent, methylated proviruses. It was recently estimated in vivo that antigenic or weak random stimulation of the host cells initiates viral replication in the HIV-1 reservoir once every 5–8 days [47]. A limited number of stimulated HIV-1 reservoir cells can survive and possibly restore the resting phenotype [48–50]. Thus, according to both proposed mechanisms the CD4^+^ T cell stimulation-mediated accumulation of 5’ LTR DNA methylation would be a rare event, increasing its probability with the time of the HIV-1 reservoir persistence.

Specifically, in the Jurkat-derived model cell line, the subset of stimulated H12 cells that harbored the reactivated provirus could be selectively destroyed by cytotoxic effects of the viral protein products, TAT, or even EGFP. In this case, the increase in 5’ LTR DNA methylation levels would not be caused by de novo CpG methylation but rather by selection for preexisting non-reactivable hypermethylated proviral 5’ LTRs that would lack expression of the viral proteins. To gain deeper insight into the situation, we compared the 5’ LTR methylation profiles of the H12 cells after consecutive series of TNF-α and PMA stimulations performed with or without selection of EGFP-negative cells. The methylation profile of 5’ LTR in the H12 cells without selection of EGFP-negative cells (mean percentage 34 % CpG methylation) did not display prevalence of hypermethylated molecules (Fig. 1d); the majority of 5’ LTR molecules contained less than half of methylated CpGs per molecule (Fig. 1e). In contrast, the H12 cells that went through the selection of EGFP-negative cells displayed the majority of highly methylated 5’ LTR molecules (Fig. 2b, middle panel) that would be anticipated in the case of selection of the preexisting hypermethylated molecules. We suggest that in the H12 model cell line, de novo methylation of the 5’ LTR after cellular stimulations with TNF-α and PMA contributes, at least in part, to the progressive accumulation of the 5’ LTR DNA methylation.

Significant alterations of the DNA methylation landscape and chromatin conformation at the genome-wide level occur during T cell stimulation, differentiation and development. Recent study showed that both DNA demethylation and de novo methylation events occurred throughout the maturation of human thymocytes although DNA demethylation prevailed over de novo methylation. Furthermore, DNA methylation changes in a particular CpG site took place generally only once during development of thymocytes and were stable in subsequent differentiation stages [51]. In mouse model, more than 1100 differentially methylated regions were identified comparing naïve and memory CD4^+^ T cells exposed to specific antigen demonstrating a link between T cell methylation status and T cell differentiation [52]. Analysis of unstimulated naïve and memory CD4^+^ cells from human healthy donors showed that CD4^+^ memory T cells were hypomethylated compared with naïve cells [53]. Another survey demonstrated that global DNA demethylation occurred rapidly (within 6 h) in mouse CD4^+^ T cells following their activation with PMA/Ionomycin [54]. On the other hand, immune activation of human naïve CD4^+^ T cells with anti-CD3/CD28 did not reveal any significant changes in DNA methylation of the promoter regions within 48 h of activation [53] despite reports suggesting that such changes occur in several genes, including IL2 and IFNγ [55–57]. Furthermore, it was showed that there is a very rapid and reversible increase in global CpG methylation after PHA/IL-2 stimulation of human PBMCs [58]. Thus, remodeling of the methylation landscape appears to occur throughout CD4^+^ T cells differentiation and following CD4^+^ T cells activation. It is believed that MBD proteins play a central role in the cellular readout of DNA methylation. Methyl-CpG binding protein 2 (MBD2) is thought of as a reader of the mCpG signature that recruits or associates with the NuRD co-repressor complex containing histone deacetylases, which in turn reinforces transcriptional silencing [59, 60].

Inhibitors of HDAC were reported to have various effects on the global DNA methylation. Inhibitor of DNA methyltransferases, decitabine, and inhibitor of HDAC, SAHA, were demonstrated to target distinct and non-overlapping regions of the genome in the human colorectal cell lines [61]. Another HDAC inhibitor, romidepsin, had essentially no effects on global DNA methylation of the human lymphoma cell lines [62]. On the other hand, treatment of porcine somatic cell nuclear transfer embryos with HDAC inhibitor oxamflatin downregulated DNMT1 expression and global DNA methylation level in two-cell-stage embryos [63]. Finally, VPA inhibition of HDAC increased global DNA methylation in human neuroblastoma cell lines [64].

Our results demonstrated that stimulation of the model cell line with TNF-α and PMA or with SAHA contributed to de novo DNA methylation of the 5’ LTR. Several studies in a human cell line or mouse model manifested an unconventional role of NF-κB as a transcriptional repressor that mediated DNA methylation by recruiting DNMT1 or DNMT3B to the target DNA sequence [65–67]. The recruitment of DNA methyltransferases via NF-κB would offer a possible mechanism for the accumulation of 5’ LTR DNA methylation after TNF-α and PMA treatment.

We demonstrated that the depletion of DNMT1, but not DNMT3B, contributed to a partial loss of 5’ LTR methylation in the 2D12 cell line. Accordingly, in the mouse model, it was suggested that DNMT1-dependent methylation activity, in contrast to DNMT3B, was essential for maintaining methylation and suppressing retrotransposon LTRs [68, 69]. We did not test the effect of DNMT3A depletion on 5’ LTR methylation. Previous study from our laboratory using avian leucosis/sarcoma virus-based reporter vector showed that absence of DNMT3A resulted only in a slight additional decrease of silencing efficiency in comparison with the knockout of DNMT3B alone and DNMT3A scored weaker than DNMT3B in silencing rescue experiments [70]. In addition, DNMT3A was established to be recruited by H3K36 me3 histone modification that was enriched in exon regions compared to introns [71, 72], and we have previously localized the HIV-1 mini-provirus in the 2D12 cell line within the intron of *UBQLN1* (ubiquilin 1) gene [29].

It was observed in CD4^+^ T cell lines and in the primary T cell model of HIV-1 latency that MBD2 is recruited to the second, but not to the first, CpG island of HIV-1 promoter. Together with MBD2, the HDAC2 was recruited to the second CpG island. Further, inhibition of DNA methylation reduced the methylation level of both CpG islands and abrogated the recruitment of MBD2 and HDAC2, resulting in partial transcriptional reactivation [37]. In our experiments, depletion of HDAC2 in the 2D12 cell line induced partial reactivation of the HIV-1 provirus by NF-κB inducers without changing DNA methylation level of the first CpG island. Consistently, in our previous work, we showed that the levels of histone 3 acetylation in the HIV-1 5’ LTR in 2D12 cell line increased 2.3-times after TNF-α and PMA stimulation [29].

We and others have shown previously that HIV-1 5’ LTR DNA methylation in combination with the chromatin structure of the latent HIV-1 5’ LTR tightly controls reactivation of the provirus. Although the latent provirus with highly methylated 5’ LTR can be reactivated, the efficiency of reactivation was lower than for the provirus with hypomethylated 5’ LTR. After the provirus reactivation in vivo, the majority of host cells are believed to be eliminated by the viral cytopathic effect or by the immune system. In fact, we detected populations of hypermethylated molecules that were present at a low frequency in the latent reservoir of HIV-1-infected individuals who were treated for long periods of time. Reactivation-resistant hypermethylated proviral molecules in the latent reservoir would thus facilitate long-term survival of the host cells and would represent the fraction of the latent reservoir with long-term stability.

Furthermore, the hypermethylated provirus could be replicated during homeostatic proliferation of cells of the HIV-1 latent reservoir [73–75]. Clonal expansion of certain proviral populations of the latent reservoir, including CD4^+^ memory cells, was shown to increase with the time spent on ART [76–78]. In the present study, after the long-term persistence of the HIV-1 reservoir, we observed populations of highly methylated molecules and of non-methylated molecules within the same patient (nos. LT1, LT5, LT10), suggesting the possibility of clonal expansion of hypermethylated 5’ LTR populations.

Importantly, an analysis by Cohn et al. [78] demonstrated that the majority of clonally expanded integrated proviruses are defective and contain mainly deletions or mutations of the 5’ LTRs. However, Maldarelli et al. [76] showed that a highly expanded clone of cells produced HIV virions in sufficient quantity to cause viremia. In our study, we did not find mutations of CpG dinucleotides, rearrangements, or polymorphisms to occur preferentially in highly methylated versus non-methylated 5’ LTR sequences from HIV-1-infected individuals.

It was observed recently that over 10 % of the integrated latent proviruses in patients could not be reactivated by stimulation of T cells harboring HIV-1. Importantly, the reactivation-resistant fraction of the latent reservoir was reported to lack inactivating mutations or promoter DNA methylation and was not integrated into transcriptionally silent regions [40]. However, in the mentioned study, only six patients who were treated for 6 years were analyzed for 5’ LTR DNA methylation [40]. Although stochastic fluctuations of transcription factors were suggested to play a role in the reactivation of the latent HIV-1 reservoir [79], we speculate that the 5’ LTR DNA methylation of the small population of latent HIV-1 molecules may further limit the reactivation capacity of the fraction of the latent reservoir.

In our previous study [29], we reported increased 5’ LTR methylation levels in memory CD4^+^ T cells of six aviremic HIV-1-infected individuals. The patients were treated for a period of 4–17 years (median 11.5 years). More recently, Blazkova et al. [39] reported that the median frequency of methylated CpGs within the HIV-1 5’ LTR was only 2.4 % (range 0–10 %). The resting CD4^+^ T cells of 11 aviremic patients who were on therapy for a median of 2.9 years (maximum 6.6 years) were analyzed in this case [39]. Both studies used the same technique of bisulfite sequencing. One of the sources of variability between the studies may lie in the different lengths of ART treatment. In the present survey, we analyzed the HIV-1 latent reservoir in two cohorts with different durations of treatment; the median treatment period was 2.3 years for the first cohort and 12.5 years for the second cohort. Intriguingly, in the cohort of patients who were treated for the shorter period of time, we detected 0 to 8 % CpG methylation in the 5’ LTR, whereas in the long-term-treated cohort, the level reached 0 to 45 % methylated CpGs. We propose that the accumulation of 5’ LTR DNA methylation may be observed in patients on ART with a long-term evolution of the latent reservoir.

Other sources of variability in the methylation levels of the HIV-1 latent reservoir could lie in the different characteristics of patients and different ART regimens. No significant difference in the plasma viral charge was detected in the present report between the group of HIV-1-infected individuals treated for up to 3 years and the long-term treated individuals (Tables S1 and S2). Patients from the study [29] showing high methylation levels of the latent HIV-1 5’ LTR were treated by an inefficient therapy regimen at the beginning. The same was true for patients from the long-term-treated cohort in our present report. We speculate that the inefficient ART regimen may influence the accumulation of DNA methylated proviruses in the reservoir indirectly, e.g., by affecting the reservoir size. However, to calculate the impact of other clinical parameters, a larger cohort of patients needs to be evaluated.

The mechanisms of HIV-1 latency maintenance are of crucial importance for therapeutic outcomes. So far, various efforts to activate the latent provirus using VPA or SAHA have been insufficient to reduce the latent reservoir size. In the present study, we focused on the role of 5’ LTR DNA methylation in long-term HIV-1 latency. We observed de novo appearance and gradual accumulation of 5’ LTR DNA methylation in the latent provirus 5’ LTR in an in vitro cell line model after successive stimulations of cells. Importantly, we confirmed the presence of limited numbers of highly methylated HIV-1 proviruses in the latent reservoir of patients after long-term persistence. We speculate that the long-term development of HIV-1 promoter methylation in the latent reservoir of infected patients contributes to reservoir persistence under ART treatment. Furthermore, we speculate that therapeutic attempts to activate the HIV-1 latent reservoir would contribute, at least in part, to an increase of 5’ LTR DNA methylation of certain latent HIV-1 proviruses resulting in an increase of reservoir stability.

## Conclusions

We have shown before that 5‘LTR DNA methylation of HIV-1 promoter stabilizes the latent state and limits the reactivation of the provirus. Here, we focused on temporal development of DNA methylation in HIV-1 promoter. We demonstrated that stimulation of model CD4^+^ T cell line with TNF-α and PMA or with SAHA contributed, at least in part, to accumulation of DNA methylation of the HIV-1 promoter. We further showed that accumulation of 5‘LTR DNA methylation occured also in the resting CD4^+^ T cells of HIV-1-infected individuals characterized by the long-term existence of the latent reservoir. Further, a limited fraction of the long-term latent reservoir within some patients contained the highly methylated HIV-1 promoters that could represent the reactivation-resistant fraction of the latent reservoir. We speculate that the stimulation of cells harboring the latent HIV-1 provirus may contribute to increase of DNA methylation level of the HIV-1 promoter and therefore may contribute to the persistence of the reservoir.

## Methods

### Ethics statement

This study was conducted according to the principles expressed in the Declaration of Helsinki. Each patient provided informed written consent to participate in this study in accordance with institutional and regulatory guidelines. The study was approved by the Ethics Committee of the Hospital Na Bulovce in Prague.

### Patients

Seropositive individuals were selected for the study on the basis of the length of ART treatment and low viral load (<320 HIV-1 RNA copies per milliliter of plasma as determined with COBAS AmpliPrep/COBAS TaqMan HIV-1 Test, version 2.0, Roche).

### Cell culture and stimulation

The H12 cell line was kindly provided by E. Verdin. The cell line was described previously [80]. The 2D12 cell line was prepared by stimulation of the H12 cell line with TNF-α and PMA and subsequent single-cell sorting of EGFP-negative cells and clonal selection [29].

Cells were grown in RPMI 1640 medium containing 2 mM L-glutamine (Sigma-Aldrich) supplemented with 10 % fetal bovine serum, 100 U/ml penicilin and 100 μg/ml streptomycin at 37 °C under a 95 % air/5 % CO_2_ atmosphere. To reactivate the HIV-1 provirus, we treated the cell lines with 10 nM PMA (Sigma-Aldrich) and 10 ng/ml TNF-α (Sigma-Aldrich) or with 0.5 μM SAHA (Sigma-Aldrich) for 24 h. Reactivation was assayed immediately after the 24 h of treatment.

### Flow cytometry analysis

We analyzed EGFP-positive and anti-CD69-phycoerythrin-labeled (Miltenyi Biotec) viable cells that were negative for staining with Hoechst 33258 (Invitrogen) at 1 μg/ml with an LSRII flow cytometer (BD Biosciences). Further analysis of flow cytometry data was carried out using FlowJo software.

EGFP-negative cells were isolated using an Influx high-speed cell sorter (BD Biosciences).

### siRNA transfection

Specific ON-TARGETplus SMARTpool siRNAs against DNMT1, DNMT3B, HDAC1 and HDAC2 genes, along with a negative control, ON-TARGETplus Non-targeting siRNAs, were purchased from Dharmacon (GE Healthcare Life Sciences). The Lipofectamine RNAiMAX transfection reagent (Life Technologies) was used for siRNA transfection. The amount of 6 × 10^5^ 2D12 cells were seeded into 6-well plates and mixed with the siRNA-complex consisting of 90 pmol of specific siRNA SMARTpool or non-targeting negative control- and the lipofectamine RNAiMAX transfection reagent. Three or 6 days after the transfection, the cells were collected to analyze the efficiency of knockdown by Western blot analysis, relative quantitative RT-PCR, or to perform TNF-α and PMA stimulations and DNA isolations. Transfection efficiency was assessed to be 40 % according to the transfection efficiency of FITC-labeled BLOCK-iT™ Fluorescent Oligo (Invitrogen).

### Western blot analysis

Thirty micrograms of total protein was loaded on 8 % SDS polyacrylamide gel and transferred to polyvinylidene difluoride membrane, which was consequently blocked in 5 % BSA. The membrane was then probed with 1:1000 dilution of Anti-DNMT1 antibody [EPR3522] (ab92314) (Abcam) or 1:250 dilution of anti-DNMT3B antibody [52A1018] (Abcam), HRP-conjugated secondary anti-rabbit antibody (Cell Signaling Technology), and anti-mouse antibody (Cell Signaling Technology). The luminescent reaction was conducted using LumiGLO (Cell Signaling Technology). Equal protein loading and transfer was verified by monoclonal Anti-γ-Tubulin, Clone GTU-88 (Sigma-Aldrich) on the same membrane.

### Quantitative RT-PCR

Total RNA was isolated from approximately 2 × 10^6^ cells using RNAzol RT (Molecular Research Center, Inc.). One microgram of total RNA was reverse-transcribed using random primers (Promega) and ProtoscriptII Reverse Transcriptase (New England BioLabs). Gene expression was then evaluated by relative quantitative RT-PCR (qRT-PCR) using the MESA GREEN qPCR Master Mix Plus for SYBR Assay (Eurogentec) in a C1000 Touch thermal cycler (Bio-Rad). See Additional file 9: Table S3 for the primer sequences. All reactions were run in triplicate, and the average Cts were used for quantitation. Signals were normalized to the corresponding RNA polymerase II, polypeptide A (*POLR2A*) housekeeping gene controls. The relative quantification of the target genes was determined using the ∆∆Ct method. The negative controls contained water instead of a template. All quantitative RT-PCRs were performed in triplicates. Data analysis was conducted with the aid of the CFX Manager software version 3.1 (Bio-Rad).

### Preparation of resting and memory CD4^+^ T cells from PBMCs of HIV-1-infected patients

Patients’ PBMCs were separated using a BD Vacutainer CPT™ Cell Preparation Tube (BD Medical). PBMCs were separated by density gradient centrifugation of blood samples. Separated PBMCs were washed twice with PBS and used for further preparation. CD4^+^ T cells were separated from PBMCs by means of CD4^+^ T Cell Isolation Kit (Miltenyi Biotec). Non-CD4^+^ cells were labeled using a cocktail of biotin-conjugated antibodies (against CD8, CD14, CD15, CD16, CD19, CD36, CD56, CD123, TCRγ/δ, and CD235a). Subsequently, non-CD4^+^ cells marked with biotin were magnetically labeled with anti-biotin microbeads. CD4^+^ T cells were isolated by depletion of magnetically labeled cells. In the second step, the activated CD4^+^ T cells were labeled for activation markers with anti-CD25-PE, anti-CD69-PE, and anti-HLA-DR-PE (all Miltenyi Biotec). Marked cells were magnetically labeled with anti-PE microbeads. Resting CD4^+^ T cells were isolated by depletion of magnetically labeled activated CD4^+^ T cells. The purity of resting CD4^+^ T cells determined by FACS analysis was greater than 95 %. Memory CD4^+^ T cells were separated from PBMCs in one step by means of a Memory CD4^+^ T cell Isolation Kit (Miltenyi Biotec). Naive CD4^+^ T cells and non-CD4^+^ T cells were magnetically labeled using a cocktail of biotin-conjugated antibodies and anti-biotin microbeads. Isolation of memory CD4^+^ T cells was achieved by depletion of magnetically labeled cells.

### Bisulfite cytosine methylation analysis

The samples of total genomic DNA from patients‘ resting or memory CD4^+^ T cells or from the H12 and 2D12 cells were isolated using proteinase K, RNase A, phenol-chloroform extraction and ethanol precipitation. Two micrograms of genomic DNA was used for bisulfite conversion using Epitect Plus DNA Bisufite Kit (Qiagen Inc.) and eluted in a total of 25 μl of water. Bisulfite-treated DNA was amplified by nested PCR specific for the 5‘LTR in a 50-μl reaction mixture. The first PCR round was performed with approximately 120 ng of genomic DNA; 2 μl from the first round was used for the second round. See Additional file 10: Table S4 for the primer sequences. The sense primers contained T and the antisense primers A instead of C in positions complementary to non-methylable C (i.e., C out of CpG dinucleotides). Cycling conditions are described in Additional file 11. At least three primary PCRs were performed for each sample to exclude amplification of one template molecule. Taking into account that approximately one resting CD4^+^ T cell out of 100–1000 contains integrated HIV-1 provirus, we placed about 10–100 proviruses into each PCR amplification. However, bisulfite treatment is known to cause significant fragmentation of DNA and the final number of template proviral molecules in one primary PCR reaction should be <10–100. Ten proviral templates represented the approximate detection threshold of bisulfite conversion followed by nested PCR as determined by dilution of the genomic DNA isolated from the H12 cell line. Non-converted DNA did not provide bands. Several non-template controls were included in each bisulfite PCR reaction. Amplification products were cloned in the pGEM-T-EasyVector System (Promega, Madison, WI) and sequenced. Analysis was performed using the Quma (Quantification tool for Methylation Analysis) software http://quma.cdb.riken.jp/. Only PCR clones with at least 95 % conversion of cytosines outside CpGs were taken into account. When more converted molecules with identical sequences were obtained, only one was used for calculation of the methylated CpG percentage to minimize the bias originating from the preferential amplification of one molecule.

### Statistical analysis

Statistical analysis of continuous data was performed using Kruskal-Wallis non-parametric test (GraphPad Prism 5.04 software), the non-paired Student’s *t* test, and Mann-Whitney test. Statistical analysis of binary data was performed using Fisher’s exact test http://www.measuringu.com/ab-calc.php. The logistic model for the dependence of 5‘LTR DNA methylation levels on time was fitted in the statistical software R. Significance of the association was tested by the standard (Wald) test. The corresponding *p* values are indicated in the figure legends.

## Additional files

Additional file 1: Figure S1. High levels of DNA methylation of proviral 5’ LTR remain stable after repeated cellular stimulation of the 2D12 cell line. Capacity to decrease 5’ LTR DNA methylation level associated with increased reactivation capacity of the highly methylated proviral promoter was assessed. Repeated stimulations of the 2D12 cell line with TNF-α and PMA were performed with or without the selection of EGFP-positive cells by FACS-sorting. In contrast to the increase of DNA methylation of the proviral 5’ LTR in the H12 cell line, the high levels of 5’ LTR DNA methylation in the 2D12 cell line did not decrease after repeated cellular stimulations neither did percentage of EGFP-positive 2D12 cells increase. At the time of each stimulation, HIV-1 provirus reactivation after 24-h of TNF-α and PMA treatment was assessed according to the percentage of EGFP-positive cells. Bisulfite sequence determined the level of 5’ LTR CpG methylation. Bisulfite sequencing of the 5’ LTR was performed at 24 days after each stimulation, when the cells were restored to the non-stimulated, steady state. (A) Latent HIV-1 provirus CpG methylation levels in the 2D12 5’ LTR sequences after repeated stimulations of cells. The percentage of methylated CpGs in the 5’ LTR was determined by bisulfite sequencing. The number of successive stimulations is depicted on the *x*-axis whereas the mean percentage of methylated CpGs is depicted on the *y*-axis. The solid line shows activations without selection of EGFP-positive cells, and the dashed line indicates activations followed by selection of EGFP-positive cells. (B) Latent HIV-1 provirus reactivation in the 2D12 cell line after repeated stimulations of cells. The 2D12 cell line was stimulated with TNF-α and PMA for 24 h, and the percentage of EGFP-positive cells was determined by FACS. The number of successive stimulations is depicted on the *x*-axis, and the percentage of EGFP-positive cells is depicted on the *y*-axis. The solid line shows activations without selection of EGFP-positive cells whereas the dashed line indicates activations followed by selection of EGFP-positive cells. (TIF 313 kb) Additional file 2: Figure S2. Proviral activation and cellular stimulation of the H12 cell line after TNF-α and PMA or SAHA treatment. The H12 cell line was stimulated for 24 h with TNF-α and PMA (upper dot plot) or with SAHA (lower dot plot). EGFP fluorescence representing provirus reactivation is depicted on *x*-axis. PE-labeled-CD69 immunofluorescence representing cellular stimulation is depicted on *y*-axis. Representative experiment is presented. (TIF126 kb) Additional file 3: Figure S3. Hypermethylation of 5’ LTR DNA in the HIV-1 latency model cell line 2D12 is maintainted by DNMT1. Latent HIV-1 provirus reactivation levels after 6 days of DNMT1 knockdown. 2D12 cells were transfected with siCTRL or with siDNMT1. The upper row of histograms represents analyses without cellular stimulation whereas the lower row represents histograms after 24 h of TNF-α and PMA stimulation. EGFP fluorescence representing provirus reactivation is depicted on *x*-axis; PE-labeled-CD69 immunofluorescence representing cellular stimulation is depicted on *y*-axis. Representative experiment is presented. *p* values were calculated by the non-paired Student’s *t* test from three independent experiments. Significance *** was assigned for *p* values <0.001. (TIF 206 kb) Additional file 4: Figure S4. mRNA expression of histone deacetylases in the 2D12 cell line. HDAC1, HDAC2, HDAC3, and HDAC8 relative mRNA levels in the non-stimulated 2D12 cells and 2D12 cells stimulated for 24 h with TNF-α and PMA were determined by qRT-PCR. mRNA expression was normalized to the expression of RNA polymerase II, polypeptide A (*POLR2A*) housekeeping gene. Data are presented as a mean ± SD of triplicates. The *p* values were calculated by the non-paired Student’s *t* test. Significance was assigned as follows: ** for *p* values <0.01, *** for *p* values <0.001. (TIF 63 kb) Additional file 5: Table S1. HIV-1-infected patients treated for up to 3 years. (PDF 162 kb) Additional file 6: Figure S5. 5’ LTR CpG methylation of the HIV-1 provirus in the latent reservoir of infected patients who were treated for up to 3 years. (A) 5’ LTR methylation profiles of HIV-1-infected individuals, nos. 1 to 15, are presented. Individual no. 3 was analyzed after 20 and 30 months of the therapy. The methylation levels are presented as a mean percentage of methylated CpGs (mCpGs) in HIV-1 promoters. An analysis of promoter molecules is shown as a linear array of open circles representing non-methylated CpG residues and closed circles representing methylated CpG residues. Each line represents one sequenced molecule of the 5’ LTR. The rectangle schematically represents the 5’ LTR region U3 with a distribution of individually analyzed CpG dinucleotides and transcription factor binding sites. (B) Levels of HIV-1 5’ LTR methylation in patients who were treated for up to 3 years. Each diamond represents one patient, numbers of patients are indicated corresponding to Additional file 5: Table S1. The time (months) spent undergoing therapy is depicted on the *x*-axis whereas the percentage of methylated CpGs in the HIV-1 5’ LTR in the latent reservoir of infected patients is depicted on the *y*-axis. The methylation levels are presented as a mean percentage of methylated CpGs in HIV-1 promoters. (TIF 539 kb) Additional file 7: Table S2. HIV-1-infected patients treated for long-term. (PDF 166 kb) Additional file 8: Figure S6. Model describing dependence of 5‘LTR DNA methylation levels in the latent reservoir of HIV-1-infected individuals on time. Patients treated for up to 3 years and long-term-treated patients were analyzed as one group. One out of the 25 analyzed HIV-1-infected persons was deleted due to its outlyingness. The odds ratio (OR) corresponding to one year difference in length of treatment was estimated to be 1.075 (*p* < 0.001). The estimated formula describing dependence of proportion [methylated CpGs] on time is logit(*p*) = −3.6910 + 0.0727 *t*, where *p* is proportion of [methylated CpGs] after *t* years of treatment. (TIF 572 kb) Additional file 9: Table S3. Primers used for qPCR reactions. (PDF 167 kb) Additional file 10: Table S4. Primers used for bisulfite-specific PCR reactions. (PDF 141 kb) Additional file 11.
**Supplementary methods.** (PDF 81 kb)

## Abbreviations

ART: antiretroviral therapy
DNMT1: DNA methyltransferase 1
DNMT3B: DNA methyltransferase 3B
HDAC: histone deacetylase
HIV-1: human immunodeficiency virus type 1
LT: long-term
LTR: long terminal repeat
MBD2: methyl-CpG binding protein 2
PBMCs: peripheral blood mononuclear cells
PMA: phorbol-12-myristate-13-acetate
POLR2A: RNA polymerase II, polypeptide A
SAHA: suberoylanilide hydroxamic acid
TNF-α: tumor necrosis factor-α
VPA: valproic acid
