# Supplementary figures and images for: Development of 5‘ LTR DNA methylation of latent HIV-1 provirus in cell line models and in long-term-infected individuals
Source: Clin Epigenetics. 2016 Feb 19;8:19. doi: 10.1186/s13148-016-0185-6 (PMC4759744; doi:10.1186/s13148-016-0185-6)

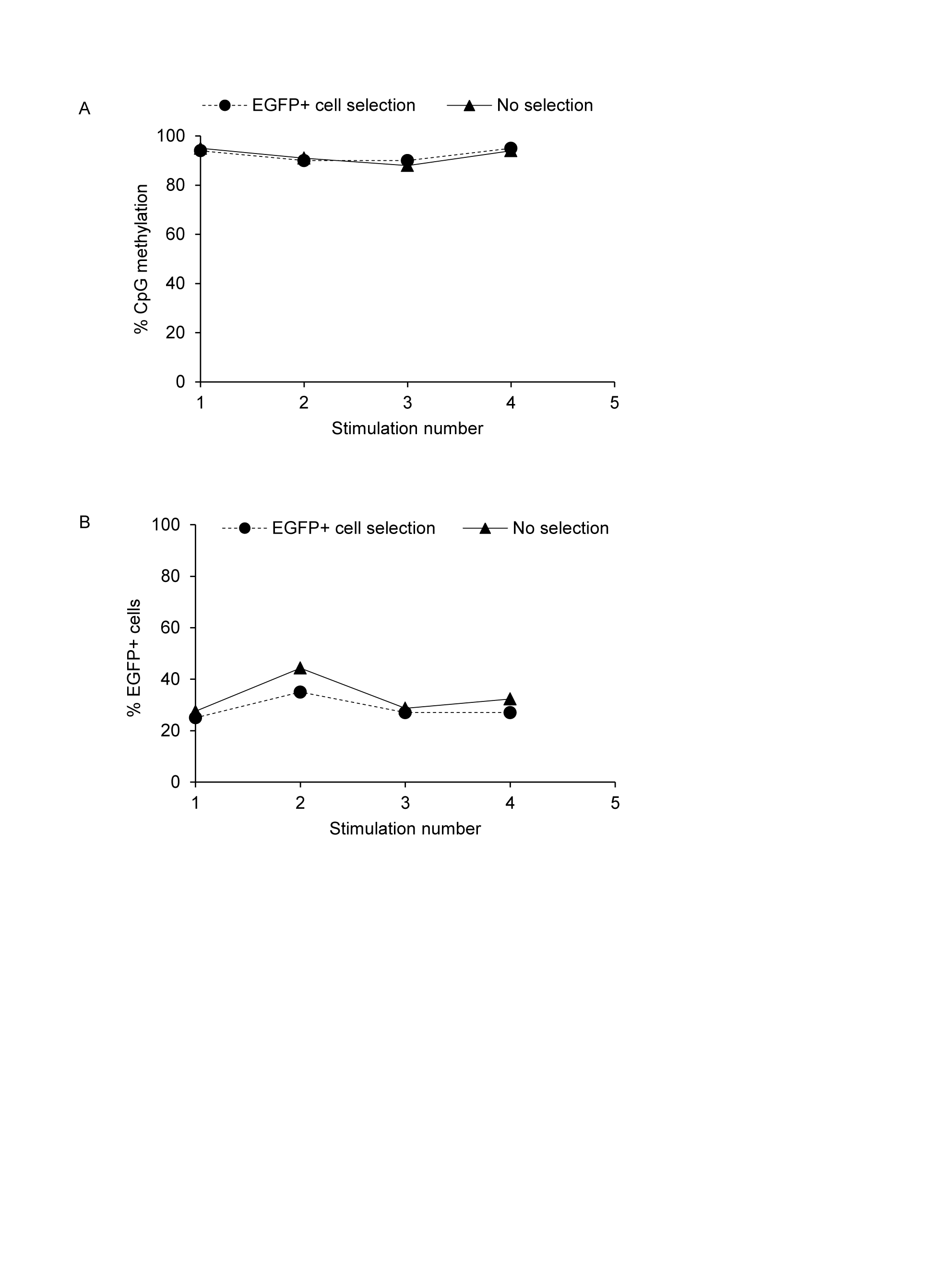

Supplement: Additional file 1: Figure S1. — High levels of DNA methylation of proviral 5’ LTR remain stable after repeated cellular stimulation of the 2D12 cell line. Capacity to decrease 5’ LTR DNA methylation level associated with increased reactivation capacity of the highly methylated proviral promoter was assessed. Repeated stimulations of the 2D12 cell line with TNF-α and PMA were performed with or without the selection of EGFP-positive cells by FACS-sorting. In contrast to the increase of DNA methylation of the proviral 5’ LTR in the H12 cell line, the high levels of 5’ LTR DNA methylation in the 2D12 cell line did not decrease after repeated cellular stimulations neither did percentage of EGFP-positive 2D12 cells increase. At the time of each stimulation, HIV-1 provirus reactivation after 24-h of TNF-α and PMA treatment was assessed according to the percentage of EGFP-positive cells. Bisulfite sequence determined the level of 5’ LTR CpG methylation. Bisulfite sequencing of the 5’ LTR was performed at 24 days after each stimulation, when the cells were restored to the non-stimulated, steady state. (A) Latent HIV-1 provirus CpG methylation levels in the 2D12 5’ LTR sequences after repeated stimulations of cells. The percentage of methylated CpGs in the 5’ LTR was determined by bisulfite sequencing. The number of successive stimulations is depicted on the x-axis whereas the mean percentage of methylated CpGs is depicted on the y-axis. The solid line shows activations without selection of EGFP-positive cells, and the dashed line indicates activations followed by selection of EGFP-positive cells. (B) Latent HIV-1 provirus reactivation in the 2D12 cell line after repeated stimulations of cells. The 2D12 cell line was stimulated with TNF-α and PMA for 24 h, and the percentage of EGFP-positive cells was determined by FACS. The number of successive stimulations is depicted on the x-axis, and the percentage of EGFP-positive cells is depicted on the y-axis. The solid line shows activations without selection of E [file 13148_2016_185_MOESM1_ESM.tif]

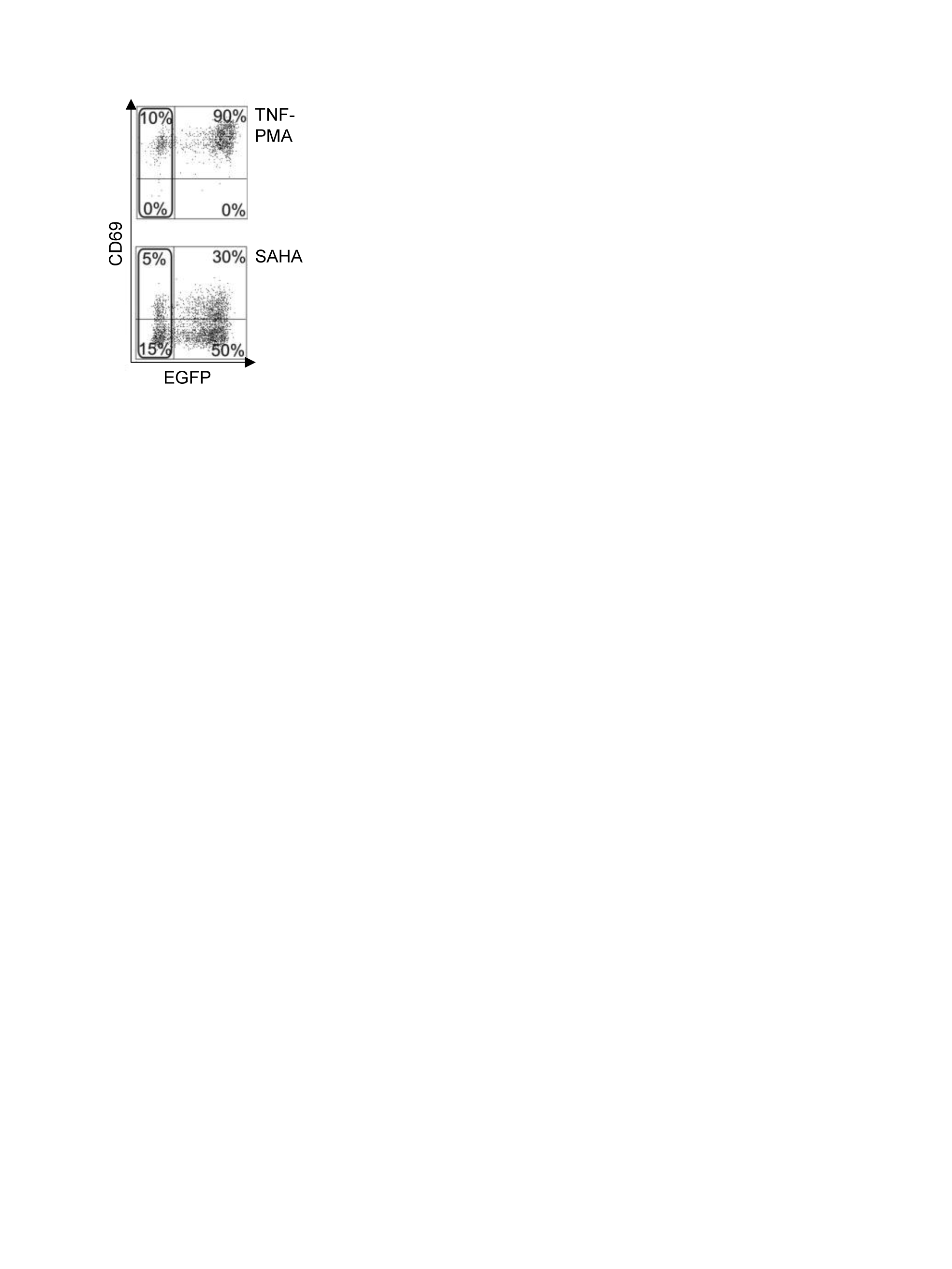

Supplement: Additional file 2: Figure S2. — Proviral activation and cellular stimulation of the H12 cell line after TNF-α and PMA or SAHA treatment. The H12 cell line was stimulated for 24 h with TNF-α and PMA (upper dot plot) or with SAHA (lower dot plot). EGFP fluorescence representing provirus reactivation is depicted on x-axis. PE-labeled-CD69 immunofluorescence representing cellular stimulation is depicted on y-axis. Representative experiment is presented. (TIF126 kb) [file 13148_2016_185_MOESM2_ESM.tif]

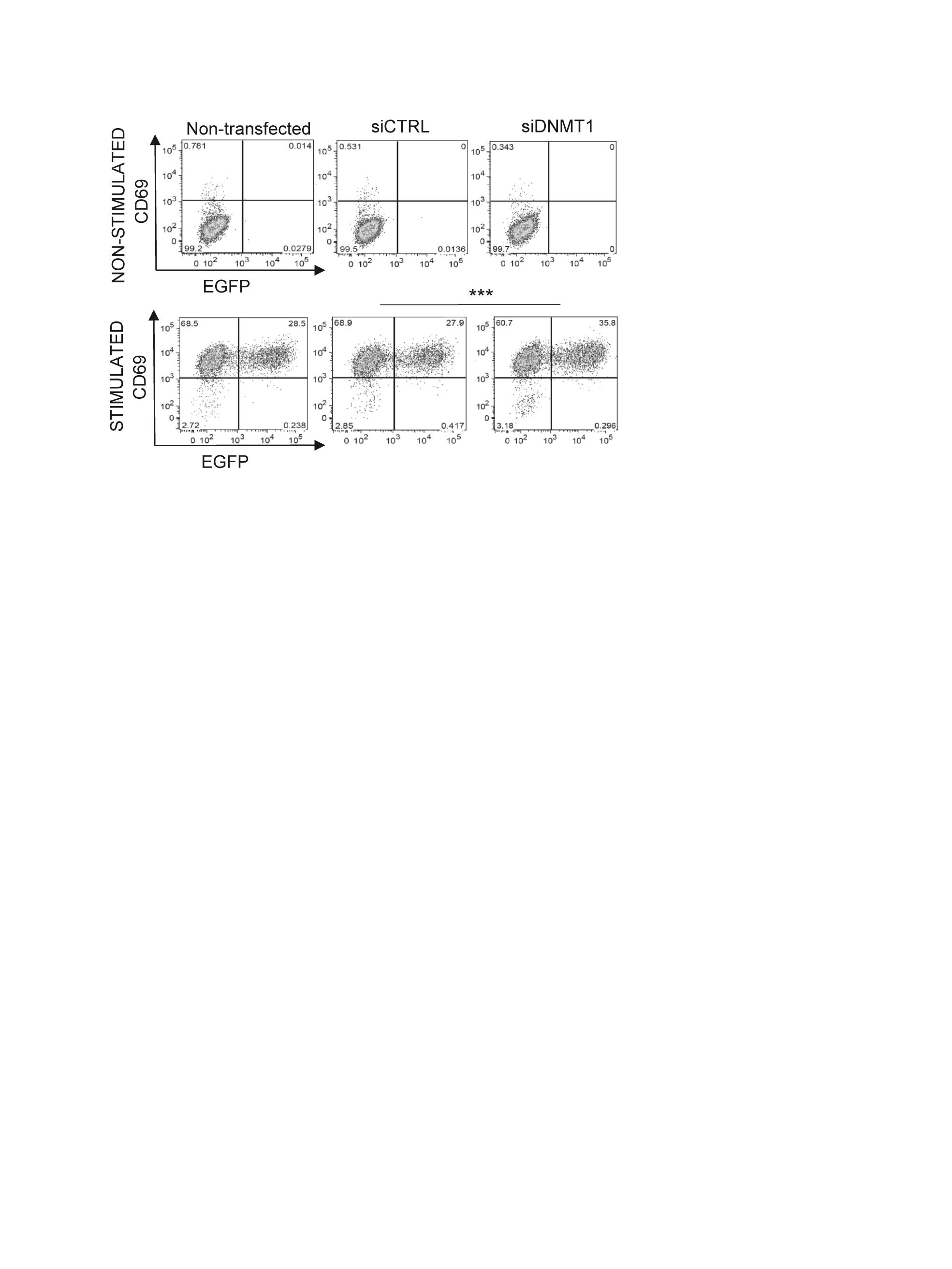

Supplement: Additional file 3: Figure S3. — Hypermethylation of 5’ LTR DNA in the HIV-1 latency model cell line 2D12 is maintainted by DNMT1. Latent HIV-1 provirus reactivation levels after 6 days of DNMT1 knockdown. 2D12 cells were transfected with siCTRL or with siDNMT1. The upper row of histograms represents analyses without cellular stimulation whereas the lower row represents histograms after 24 h of TNF-α and PMA stimulation. EGFP fluorescence representing provirus reactivation is depicted on x-axis; PE-labeled-CD69 immunofluorescence representing cellular stimulation is depicted on y-axis. Representative experiment is presented. p values were calculated by the non-paired Student’s t test from three independent experiments. Significance *** was assigned for p values <0.001. (TIF 206 kb) [file 13148_2016_185_MOESM3_ESM.tif]

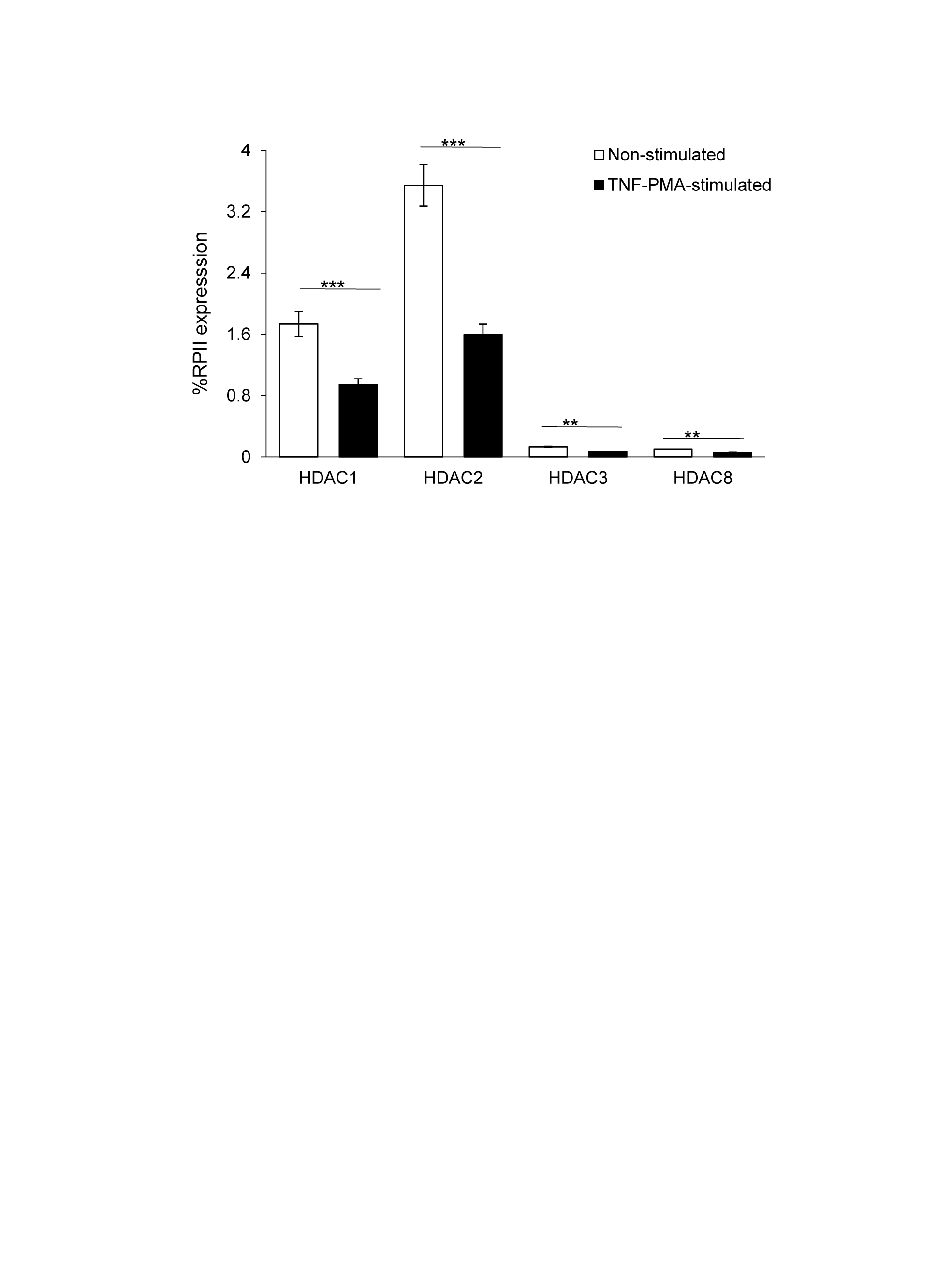

Supplement: Additional file 4: Figure S4. — mRNA expression of histone deacetylases in the 2D12 cell line. HDAC1, HDAC2, HDAC3, and HDAC8 relative mRNA levels in the non-stimulated 2D12 cells and 2D12 cells stimulated for 24 h with TNF-α and PMA were determined by qRT-PCR. mRNA expression was normalized to the expression of RNA polymerase II, polypeptide A (POLR2A) housekeeping gene. Data are presented as a mean ± SD of triplicates. The p values were calculated by the non-paired Student’s t test. Significance was assigned as follows: ** for p values <0.01, *** for p values <0.001. (TIF 63 kb) [file 13148_2016_185_MOESM4_ESM.tif]

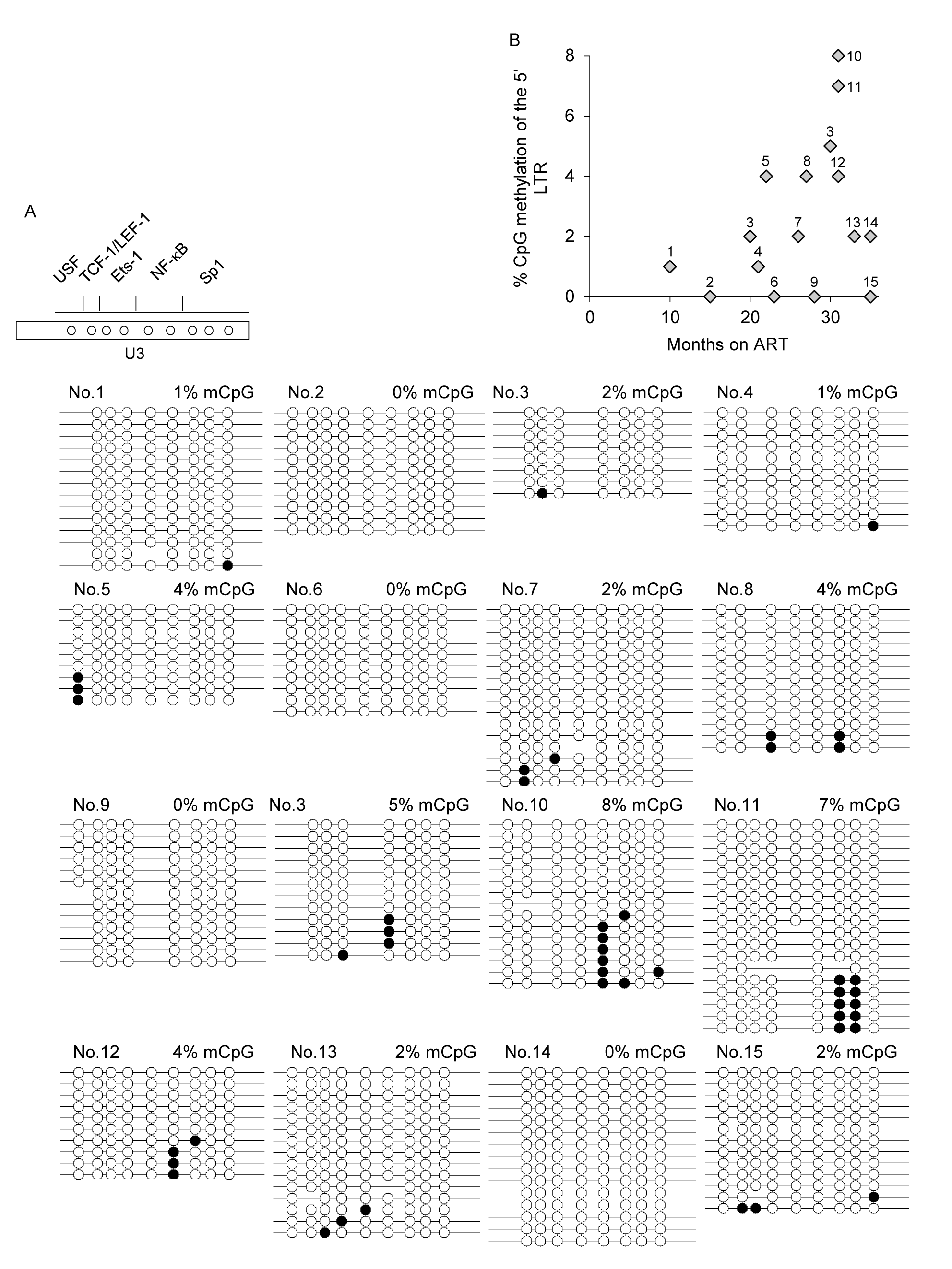

Supplement: Additional file 6: Figure S5. — 5’ LTR CpG methylation of the HIV-1 provirus in the latent reservoir of infected patients who were treated for up to 3 years. (A) 5’ LTR methylation profiles of HIV-1-infected individuals, nos. 1 to 15, are presented. Individual no. 3 was analyzed after 20 and 30 months of the therapy. The methylation levels are presented as a mean percentage of methylated CpGs (mCpGs) in HIV-1 promoters. An analysis of promoter molecules is shown as a linear array of open circles representing non-methylated CpG residues and closed circles representing methylated CpG residues. Each line represents one sequenced molecule of the 5’ LTR. The rectangle schematically represents the 5’ LTR region U3 with a distribution of individually analyzed CpG dinucleotides and transcription factor binding sites. (B) Levels of HIV-1 5’ LTR methylation in patients who were treated for up to 3 years. Each diamond represents one patient, numbers of patients are indicated corresponding to Additional file 5: Table S1. The time (months) spent undergoing therapy is depicted on the x-axis whereas the percentage of methylated CpGs in the HIV-1 5’ LTR in the latent reservoir of infected patients is depicted on the y-axis. The methylation levels are presented as a mean percentage of methylated CpGs in HIV-1 promoters. (TIF 539 kb) [file 13148_2016_185_MOESM6_ESM.tif]

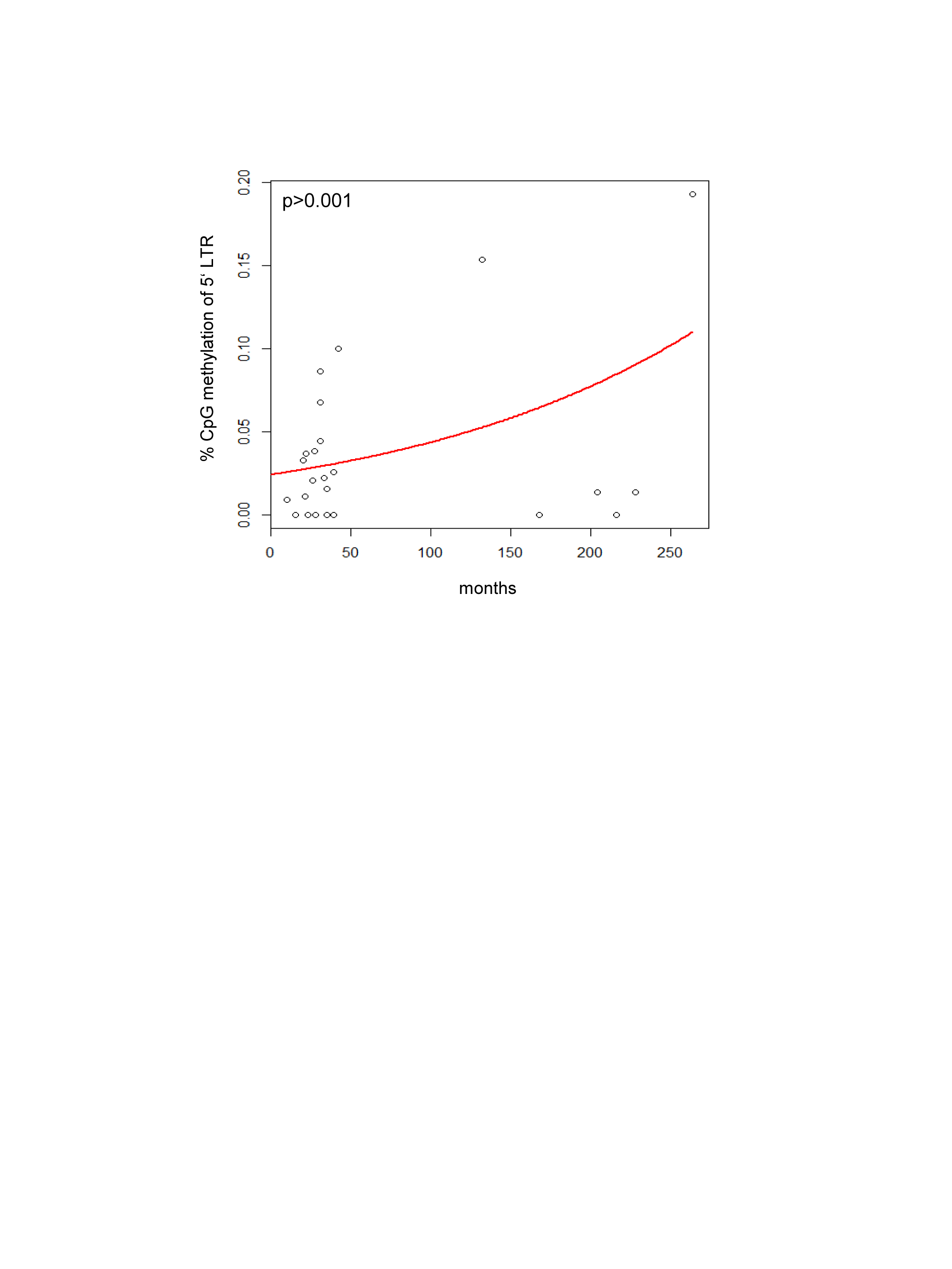

Supplement: Additional file 8: Figure S6. — Model describing dependence of 5‘LTR DNA methylation levels in the latent reservoir of HIV-1-infected individuals on time. Patients treated for up to 3 years and long-term-treated patients were analyzed as one group. One out of the 25 analyzed HIV-1-infected persons was deleted due to its outlyingness. The odds ratio (OR) corresponding to one year difference in length of treatment was estimated to be 1.075 (p < 0.001). The estimated formula describing dependence of proportion [methylated CpGs] on time is logit(p) = −3.6910 + 0.0727 t, where p is proportion of [methylated CpGs] after t years of treatment. (TIF 572 kb) [file 13148_2016_185_MOESM8_ESM.tif]
