# Supplementary material for: Development of 5‘ LTR DNA methylation of latent HIV-1 provirus in cell line models and in long-term-infected individuals
Source: Clin Epigenetics. 2016 Feb 19;8:19. doi: 10.1186/s13148-016-0185-6 (PMC4759744; doi:10.1186/s13148-016-0185-6)
Supplement: Additional file 5: Table S1. — HIV-1-infected patients treated for up to 3 years. (PDF 162 kb) [file 13148_2016_185_MOESM5_ESM.pdf]

S1 Table. HIV-1-infected patients treated for up to three years.

No.	Sex	Infected from	On Therapy from	Therapy	Analysis after months of ART	5' LTR CpG (%)	Plasma charge (copies/ /ml)*	$CD4^+/mm^3$
**1**	M	2011	April, 2011	LPV/r+TDF+FTC	10	1	46	205
**2**	M	2011	December, 2011	RAL+TDF+FTC	15	0	0	488
**3**	M	2010	February, 2011	EFV+ABC+3TC	20	2	0	690
**4**	M	2011	January, 2012	LPV/r+FTC+TDF	21	1	53	608
**5**	M	2010	August, 2012	TDF+FTC+RAL	22	4	0	1086
**6**	M	2010	February, 2012	ATV/r+TDF+FTC	23	0	61	912
**7**	M	2007	February, 2012	RAL+TDF+FTC	26	2	7180	753
**8**	M	2011	April, 2012	EFV+TDF+FTC	27	4	0	788
**9**	M	2001	January, 2012	LPV/r+TDF+FTC	28	0	21	247
**3**	M	2010	January, 2011	EFV+ABC+3TC	30	5	0	593
**10**	M	2010	March, 2011<br>April, 2013 Up to analysis	FTC+TDF+EFV<br>FTC+TDF+RAL	31	8	60	872
**11**	M	2007	April, 2011	EFV+TDF+FTC	31	7	36	459
**12**	M	2010	August, 2011	LPV/r+TDF+FTC	31	4	0	1076

No.	Sex	Infected from	On Therapy from	Therapy	Analysis after months of ART	5' LTR CpG (%)	Plasma charge (copies/ /ml)*	$CD4^+/mm^3$
**13**	M	2011	October, 2011	LPV/r+TDF+FTC	33	2	<20	293
**14**	M	2010	April, 2011	EFV+TDF+FTC	35	0	<20	317
			April, 2013 Up to analysis	RAL+TDF+FTC				
**15**	M	2009	August, 2011	3TC+AZT+LPV/r	35	2	41	678
			June, 2012 Up to analysis	RAL+TDF+FTC				

LPV/r, Lopinavir/Ritonavir; TDF, Tenofovir; FTC Emtricitabine; RAL, Raltegravir; EFV, Efavirenz; ABC, Abacavir; 3TC, Lamivudine; ATV/r, Atazanavir/Ritonavir.
Patients No. 10, 14, 15 were treated with two different treatment regimens, the rest of patients were maintained on the same treatment regiment from the beginning of therapy up to the date of analysis. Patient 3 was analyzed at 20 and at 30 months after starting the therapy.
* No significant difference in the plasma viral charge was detected between the group of HIV-1-infected individuals treated for up to three years and the long-term treated individuals ($p>0.0769$).
