# Supplementary material for: Development of 5‘ LTR DNA methylation of latent HIV-1 provirus in cell line models and in long-term-infected individuals
Source: Clin Epigenetics. 2016 Feb 19;8:19. doi: 10.1186/s13148-016-0185-6 (PMC4759744; doi:10.1186/s13148-016-0185-6)
Supplement: Additional file 7: Table S2. — HIV-1-infected patients treated for long-term. (PDF 166 kb) [file 13148_2016_185_MOESM7_ESM.pdf]

S2 Table. HIV-1-infected patients treated for long-term.

<table>
<tr><th>No.</th><th>Sex</th><th>Infected from</th><th>On Therapy From</th><th>Therapy</th><th>Analysis after years of ART</th><th>5‘ LTR CpG (%)</th><th>Plasma charge (copies/ /ml)*</th><th>$CD4^{+}/mm^{3}$</th></tr>
<tr><td>LT1</td><td>M</td><td>2007</td><td>April, 2011</td><td>AZT+3TC+TDF</td><td>3</td><td>45</td><td>0</td><td>454</td></tr>
<tr><td>LT2</td><td>M</td><td>2010</td><td>November, 2011</td><td>LPV/r+TDF+FTC</td><td>3.25</td><td>0</td><td>0</td><td>986</td></tr>
<tr><td rowspan="2">LT3</td><td rowspan="2">M</td><td rowspan="2">2009</td><td>April, 2011</td><td>3TC+AZT+LPV/r</td><td rowspan="2">3.25</td><td rowspan="2">3</td><td rowspan="2"><20</td><td rowspan="2">967</td></tr>
<tr><td>June, 2012 Up to analysis</td><td>RAL+TDF+FTC</td></tr>
<tr><td>LT4</td><td>M</td><td>2010</td><td>September, 2010</td><td>AZT+3TC+TDF</td><td>3.5</td><td>10</td><td>0</td><td>642</td></tr>
<tr><td>LT5</td><td>M</td><td>2002</td><td>April, 2003</td><td>AZT+3TC+LPV/r</td><td>11</td><td>13</td><td>0</td><td>490</td></tr>
<tr><td rowspan="2">LT6</td><td rowspan="2">F</td><td rowspan="2">1995</td><td>2000</td><td>AZT+3TC+LPV/r</td><td rowspan="2">14</td><td rowspan="2">0</td><td rowspan="2"><20</td><td rowspan="2">1137</td></tr>
<tr><td>2003 Up to analysis</td><td>AZT+SQV+LPV/r</td></tr>
<tr><td rowspan="5">LT7</td><td rowspan="5">M</td><td rowspan="5">1996</td><td>1997</td><td>AZT+ddI+RTV</td><td rowspan="5">17</td><td rowspan="5">1</td><td rowspan="5"><20</td><td rowspan="5">944</td></tr>
<tr><td>1997</td><td>AZT+ddI+IDV</td></tr>
<tr><td>2000</td><td>AZT+ddI+IDV+RTV</td></tr>
<tr><td>2001</td><td>AZT+ddI+EFV</td></tr>
<tr><td>2006 Up to analysis</td><td>FTC+TDF+NVP</td></tr>
</table>

No.	Sex	Infected from	On Therapy From	Therapy	Analysis after years of ART	5‘ LTR CpG (%)	Plasma charge (copies/ /ml)*	$CD4^{+}/mm^{3}$
**LT8**	M	1995	November, 1996	SQV+AZT+ddC	18 years	0	0	1270
			March, 1997	SQV+AZT+3TC				
			March, 1999	IDV+ 3TC+d4T				
			November, 2000	IDV+3TC+AZT				
			April, 2001	IDV+3TC+ABC				
			May, 2007 Up to analysis	LPV/r+FTC+TDF				
**LT9**	F	1990	1995	AZT+3TC	19	1	<20	438
			1997	d4T+ddI+IDV				
			1999	d4T+ddI+IDV+RTV				
			2005	3TC+ABV+LPV				
			2006 Up to analysis	RAL+TDF+FTC				
**LT10**	M	1990	1992	ddI	22	19	320	660
			1997	AZT+3TC+IDV				
			2000	AZT+3TC+IDV+RTV				
			2001	3TC+ABV+EFV				
			2001 Up to analysis	3TC+ABC+NVP				

AZT, Zidovudine (Azidothymidine); 3TC, Lamivudine; TDF, Tenofovir; LPV/r, Lopinavir/Ritonavir; FTC, Emtricitabine; RAL, Raltegravir; SQV, Saquinavir, ddI, Didanosine, RTV, Ritonavir, IDV, Indinavir, EFV, Efavirenz, NVP, Nevirapine, ddC, Zalcitabine, d4T, Stavudine, ABC, Abacavir, LPV, Lopinavir.

Patients No. LT3, LT6, LT7, LT8, LT9, LT10 were treated with two or more treatment regimens, patients No. LT1, LT2, LT4, LT5 were maintained on the same treatment regiment from the beginning of therapy up to the date of analysis.
* No significant difference in the plasma viral charge was detected between the group of HIV-1-infected individuals treated for up to three years and the long-term treated individuals ($p>0.0769$).
