# Supplementary material for: Development of 5‘ LTR DNA methylation of latent HIV-1 provirus in cell line models and in long-term-infected individuals
Source: Clin Epigenetics. 2016 Feb 19;8:19. doi: 10.1186/s13148-016-0185-6 (PMC4759744; doi:10.1186/s13148-016-0185-6)
Supplement: Additional file 9: Table S3. — Primers used for qPCR reactions. (PDF 167 kb) [file 13148_2016_185_MOESM9_ESM.pdf]

S3 Table. Primers used for qPCR reactions

Name of primer	Sequence of primer
DNMT1-sense	5’-GAAGGGAGACGTGGAGATG-3’
DNMT1-antisense	5’-ATGGAGCGCTTGAAGGAGAC-3’
DNMT3B-sense	5’-GCCGGCTCTTCTTCGAATTT-3’
DNMT3B-antisense	5’-GAAGTATCGGGCCCTGTGAG-3’
HDAC1-sense	5’-CGAATCCGCATGACTCATAA-3’
HDAC1-antisense	5’-TCTCTGCATCTGCTTGCTGT-3’
HDAC2-sense	5’-TGGTGTCCAGATGCAAGCTA-3’
HDAC2-antisense	5’-TGATCAGCCACATTTCTTCG-3’
HDAC3-sense	5’-TGCAAGGCTTCACCAAGAGT-3’
HDAC3-antisense	5’-AACTTCTTGGCATGGTGCAG-3’
HDAC8-sense	5’-TCTGCCAAACCCTTTTCCTT-3’
HDAC8-antisense	5’-TCCAGTTCCTGCTCCTCTGA-3’
POLR2A- sense	5’-GCACCACGTCCAATGACAT-3’
POLR2A- antisense	5’-GTGCGGCTGCTTCCATAA-3’
