# Supplementary material for: Development of 5‘ LTR DNA methylation of latent HIV-1 provirus in cell line models and in long-term-infected individuals
Source: Clin Epigenetics. 2016 Feb 19;8:19. doi: 10.1186/s13148-016-0185-6 (PMC4759744; doi:10.1186/s13148-016-0185-6)
Supplement: Additional file 10: Table S4. — Primers used for bisulfite-specific PCR reactions. (PDF 141 kb) [file 13148_2016_185_MOESM10_ESM.pdf]

S4 Table. Primers used for bisulfite-specific PCR reactions.

Name of primer	Sequence of primer
MB	5‘-GGTAGAATTATATATTAGGGTTAGGGATT-3‘<br>nucleotides [nt] 81 to 110, sense
MH	5‘-CACCCATCTCTCTCCTTCTAACCTC-3‘<br>[nt] 772 to 796, antisense
MC	5‘-AGAGAAGGTAGAAGAAGTTAATGAAGGAGA-3‘<br>[nt] 161 to 190, sense
MG	5‘-AAAAAACTCCTCTAATTTCCCTTT-3‘<br>[nt] 663 to 686, antisense
MC-50	5‘-TAGATATTTATTGATTTTTGGATGGTG-3‘<br>[nt] 110 to 136, sense
MG_deg	5‘-AAAAAACTCCTCTAATTTYHCTTTC-3‘<br>[nt] 662 to 686, antisense
BIS-5LTR-FW	5‘-AGTGTTAGTGTGGAGGTTTGATA-3‘<br>[nt] 248 to 270, sense
BIS-5LTR-RV	5‘-CAAAAAAACCCAATACAAACAAAAAAC-3‘<br>[nt] 438 to 464, antisense
Env-FW	5‘-AGTTTTGTTTTTTGGGTTTTTG-3‘<br>[nt] 7140 to 7161, sense
Env-RVa	5‘-ATCTTTCCACAACCAAAATTCT-3‘<br>[nt] 7327 to 73418, antisense
Env-RVb	5‘-CCTCAATAACCCTCAACAAATTA-3‘<br>[nt] 7251 to 7273, antisense
