# Supplementary material for: Development of 5‘ LTR DNA methylation of latent HIV-1 provirus in cell line models and in long-term-infected individuals
Source: Clin Epigenetics. 2016 Feb 19;8:19. doi: 10.1186/s13148-016-0185-6 (PMC4759744; doi:10.1186/s13148-016-0185-6)
Supplement: Additional file 11. — Supplementary methods. (PDF 81 kb) [file 13148_2016_185_MOESM11_ESM.pdf]

Supplementary Methods

PCR after bisulfite treatment

All PCR reactions were performed in a deep-well block.

Bisulfite-treated DNA was amplified by nested PCR in a 50-μl reaction mixture containing 50 mM Tris-HCl (pH 9.2), 2.5 mM $MgCl_2$, each dNTP at 200 μM, 320 nM each primer and 1U of Platinum Taq Polymerase (Invitrogen). Two μg of HotStart-IT Binding Protein (Affymetrix) was added to the first PCR round for analysis of patients‘ DNA.

PCR reactions to analyze 5‘ LTR in the cell lines were performed with the following primers: MB and MH for the first round, and MC and MG for the second round.

PCR reactions to analyze 5‘ LTR from patients‘ DNA were performed with the following primers: MC-50 and MH for the first round or MG_deg instead of MH for the first round, and BIS-5LTR-FW and BIS-5LTR-RV for the second round. Using the BIS-LTR-FW together with BIS-LTR-RV in the first round of PCR never provided bands when using patients‘ DNA.

PCR reactions to analyze env region from patients‘ DNA were performed with the following primers: env-FW and env-RVa for the first round, and env-FW and env-RVb for the second round.

Cycling conditions for analysis of bisulfite-treated DNA from the cell lines were equal for the first and second PCR round:

40 cycles, 94 ^{o}C for 20 s, 58 ^{o}C for 50 s, and 72 ^{o}C for 60 s.

Cycling conditions for analysis of bisulfite-treated DNA from HIV-1-infected individuals (5‘ LTR and env), first PCR round:

20 cycles, 94 ^{o}C for 15 s, annealing for 90 s, 72 ^{o}C for 60 s,

(annealing starts at 65 ^{o}C, annealing temperature decreases by 1 ^{o}C after each two cycles until 56 ^{o}C),

20 cycles, 94 ^{o}C for 15 s, 57 ^{o}C for 90 s and 72 ^{o}C for 60 s.

Cycling conditions for analysis of bisulfite-treated DNA from HIV-1-infected individuals, second PCR round:

20 cycles, 94 ^{o}C for 15 s, 58 ^{o}C for 90 s, 72 ^{o}C for 60 s,

20 cycles, 94 ^{o}C for 15s, 65 ^{o}C for 90 s, 72 ^{o}C for 60 s.
